# Transferability of Buckingham Parameters for Short-Range Repulsion between Topological Atoms

**DOI:** 10.1021/acs.jpca.4c02048

**Published:** 2024-05-28

**Authors:** Jaiming J. K. Chung, Matthew L. Brown, Paul L. A. Popelier

**Affiliations:** Department of Chemistry, The University of Manchester, Oxford Road, Manchester M13 9PL, Great Britain

## Abstract

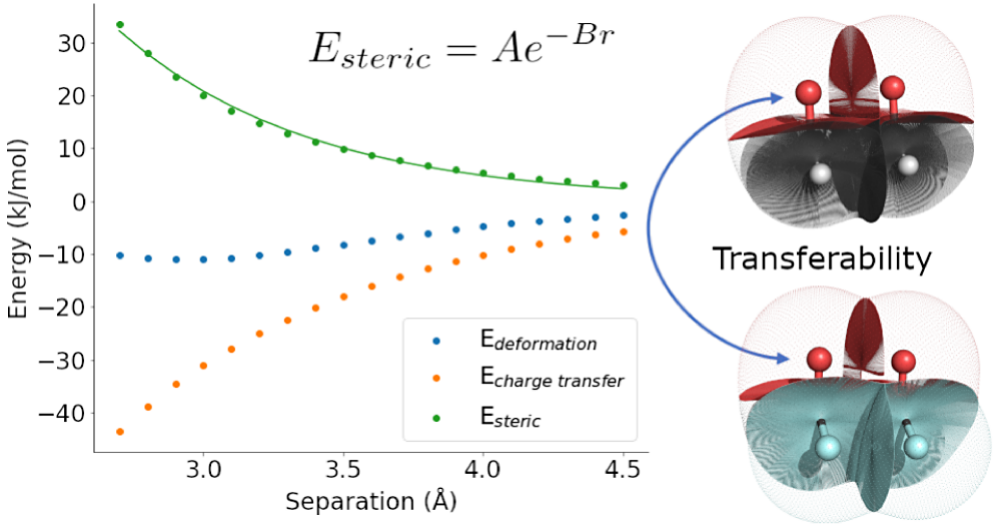

The repulsive part of the Buckingham potential, with parameters *A* and *B*, can be used to model deformation energies and steric energies. Both are calculated using the interacting quantum atom energy decomposition scheme where the latter is obtained from the former by a charge-transfer-based energy correction. These energies relate to short-range interactions, specifically the deformation of electron density and steric hindrance, respectively, when topological atoms approach each other. In this work, we calculate and fit the energies of carbonyl carbon, carbonyl oxygen, and, where possible, amine nitrogen atoms to the repulsive part of the Buckingham potential for 26 molecules. We find that while the steric energies of all atom pairs studied display exponential behavior with respect to distance, some deformation energy data do not. The obtained parameters are shown to be transferable by calculating root-mean-square errors of fitted potentials with respect to energy data of the same atom in, as far as possible, all other molecules from our data set. We observed that 36% and 10% of these errors were smaller than 4 kJ mol^–1^ for steric and deformation energy, respectively. Thus, we find that steric energy parameters are more transferable than deformation energy parameters. Finally, we speculate about the physical meaning of the *A* and *B* parameters and the implications of the strong exponential and exponential-linear piecewise relationships that we observe between them.

## 1 Introduction

Short-range repulsion refers to intermolecular forces arising from interelectronic repulsion due to the Pauli exclusion principle. These forces are important for determining atomic spacing in solids, liquids, and gases and can dominate when molecules are separated by distances of a few Ångström, which can occur at high pressures.^1^ If these forces did not exist or if they increased in strength slower than attractive forces with decreasing distance, atoms would collapse into each other, and the Universe as we know it would not exist. It is worth pointing out that, strictly speaking, the Pauli repulsive force is not a real physical force in the sense of being transmitted by bosons as fundamental forces are. Instead, it is an effect due to the antisymmetric character of an overall wavefunction keeping same-spin electrons apart.

Intermolecular forces are important in molecular dynamics (MD), which simulates the movement of atoms to predict the behavior and properties of chemical systems. MD can be performed using *ab initio* methods by programs such as the Vienna *ab initio* Simulation Package (VASP)^2^ and CP2K,^3^ although these come with considerable computational expense. Classical force fields offer an alternative at a much lower computational cost. In these force fields, energy is typically represented as a sum of simple energy terms that describe bonded and nonbonded interactions. The mathematical shape of these energy terms and the values for the parameters they contain differ between force fields, such as AMBER,^4^ CHARMM,^5^ and GROMOS.^6^ Force fields using exponential functions for nonelectrostatic nonbonded interactions have also been proposed,^7,8^ and the current work focuses on this part of a force field.

The construction of classical force fields invariably introduces an *a priori* mathematical expression for each type of energy contribution, typically with a tenuous connection to quantum mechanics, if any. Second, the construction assumes its own atom types and imposes some kind of energy partitioning, again without much of a link to quantum mechanics. However, it is possible to start from scratch and embrace an energy partitioning method upfront and let it provide the atomic energies from which to build a novel force field. A good candidate for this energy partitioning is the topological energy decomposition called interacting quantum atoms (IQA)^9^ because of its minimality (not to be confused with simplicity), as explained in the preface of ref. (10).

Our FFLUX^11^ force field is built on the atomic energies that IQA provides. The latter is a generalization^12^ of the virial-based energy partitioning of the quantum theory of atoms in molecules (QTAIM).^13^ QTAIM provides atomic multipole moments to FFLUX such that its energy treatment can be completed at both short and long range. Already^14^ in 2007, machine learning played a role in the construction of FFLUX,^11,15^ further bridging the gap between force field and *ab initio* methods. More recently, FFLUX is being used to simulate liquid water^16^ and formamide crystals^17^ accurately. However, in these calculations, the simple potentials that quantify repulsion and dispersion in classical force fields were still present. Yet, this state of the art is transient, as our preliminary work shows a route to ultimately obtain repulsion and dispersion from IQA energies rather than potentials external to IQA.

In order to understand the motivation for the work presented here, the strategy behind the development of our FFLUX^11^ force field needs to be explained first. The long-term goal of FFLUX is to provide a reliable and future-proof potential energy function to fuel the MD of peptides in aqueous solution. We believe that the key to future-proof success is to guarantee that each energy term is “what it is.” This means, for example, that the electrostatic energy between atoms does not contain any other types of energy; moreover, it must be well-defined at in any range (even between bonded atoms right next to each other). Conversely, classical force fields are known to sometimes absorb nonelectrostatic effects into the values of their (supposedly electrostatic) point charges as is the case^18^ for TIP5P, for example. Here, the force field suffers from energy terms that are not what they are meant to be, which weakens the force field’s architecture and stifles sustainable progress in its development.

At medium and long range, IQA’s interatomic electrostatic energy can be successfully expanded^19^ into a multipolar series, the convergence^20^ and accuracy of which have been carefully monitored.^21^ IQA clearly defines three more unambiguous types of energy terms: intra-atomic (including kinetic energy), exchange, and correlation energies. As reviewed^22^ fairly recently the latter has the potential to replace the Dn dispersion scheme^23^ were it not for its currently prohibitive computational cost. Second, the interatomic IQA exchange energies find a nice chemical interpretation in quantifying^24^ the degree of covalency of bonds, including “noncovalent” interactions. Importantly, these energies were successfully linked^25^ to experimental NMR *J*-coupling constants^3^*J*(H,H’). Third and finally, the intra-atomic energies (including both potential and kinetic energy) were for the first time^26^ interpreted as (short-range) steric repulsion in 2016 by Wilson and Popelier. These energies were then successfully fitted to the Buckingham potential, such that IQA made contact with an established body of research on intermolecular repulsive potentials. The purpose of the abovementioned interpretative studies is to show the chemical validity of IQA as a single, universal source of energy terms. The main question of the current work is whether the parameters fitted to the Buckingham potential have a chemical meaning.

An important factor for the success of a force field is how it handles intermolecular interactions, usually represented by a sum of electrostatic, repulsive, and dispersive terms. In many force fields, short-range repulsion is modeled using an *r*^–12^ term that appears in the Lennard-Jones potential,^27^ which is a specific form of the more general *r*^–*n*^ term in the Mie potential.^28^ However, we showed^26^ that IQA provides atomic energies that fit better an exponential function (i.e., Buckingham potential) rather than the inverse power law of Lennard-Jones. This is pleasing news because the former is generally^29^ considered to be more accurate, and thus IQA provides accurate data. The shape of this Buckingham or Born- Mayer potential is
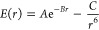
1where *E* is the potential energy, *r* is the distance between two interacting atoms, and *A*, *B*, and *C* are parameters. The exponential term is a repulsive term, while the *r*^–6^ term is an attractive term. Since this work focuses on repulsive interactions, we omit the attractive *r*^–6^ in this work.

Previous work^30^ has paid special attention to the IQA intra-atomic energies of hydrogen atoms, which lack core electrons. However, in heavier atoms, core electrons consistently provide repulsion when compressed. That work provided a deeper understanding of the energetics of steric effects, alongside a method to quantify them. However, this work already pointed out a vulnerability of this method, as exposed by hydrogen atoms. They are susceptible to changes in atomic charge because their electron density is provided entirely by valence electrons. Other researchers^31^ took this idea further and proposed a charge transfer correction to define a steric energy. This correction subtracts an approximation of charge transfer effects from the deformation energy used to define the steric energy, as explained in Section 2.2. This corrected steric energy better represents steric hindrance and has since been used to study E2 and S_N_2 reactions.^32^ In that capacity, it recovers trends expected by chemical intuition, thereby linking quantum chemistry and organic chemistry.

Another chemical concept is transferability, or the idea that the behavior of molecules depends on their constituent atoms and functional groups, which behave similarly between molecules. That is, a carbon atom will not behave like an oxygen atom and *vice versa*. However, an aliphatic sp^2^ carbon in one carbonyl, for example, should behave like an aliphatic sp^2^ carbon atom in another carbonyl. In the context of the current work, transferability refers to the ability to use the parameters for modeling a specific interaction to also model similar interactions in different molecules. Transferability of the *A* and *B* parameters implies energetic transferability of atoms, a feature of QTAIM, which provides the basis for IQA. Interestingly, the success of fragmentation methods for molecular property prediction cannot always be attributed to energetic transferability of atoms: in some cases, high accuracy is actually due to cancellation of errors.^33^ Practically, transferability can be used to define atom types^34^ as in classical force fields, speed up simulations without significantly decreasing quality, and potentially train machine learning models. Likewise, the *A* and *B* parameters having physical meaning can allow for shortcuts when calculating values for new molecules and can be useful for linking quantum chemical energies to chemical intuition.

Transferability of parameters also implies a physical meaning. For example, the Lennard-Jones parameters, σ and ε, which are transferable,^35^ respectively correspond to atomic size and the potential’s well-depth. The latter are both clear physical concepts. Moreover, σ and ε can be easily marked on the graph of potential energy against distance, where *V*(σ) = 0, while the minimum value of *V* is -ε. Currently, the nature of the Buckingham *A* and *B* parameters is less clear. Roughly speaking, *A* controls the vertical dilation of the function, while *B* controls the horizontal dilation. Both parameters affect the gradient and hence the force due to short-range repulsion, which would be a key component of a classical force field using a Buckingham potential. Thus, *A* is related to the strength of short-range repulsion, while *B* is related to how the repulsion decays with distance.

In the current work, we show that the Buckingham parameters, which are derived from fits of the IQA deformation energy and steric energy (see Section 2.2), are transferable. Results presented here show interesting behavior that both hints at a deeper physical meaning of these parameters and confirms the importance of the abovementioned steric correction.

## 2 Methods

### 2.1 Quantum Theory of Atoms in Molecules

**Figure 1:**
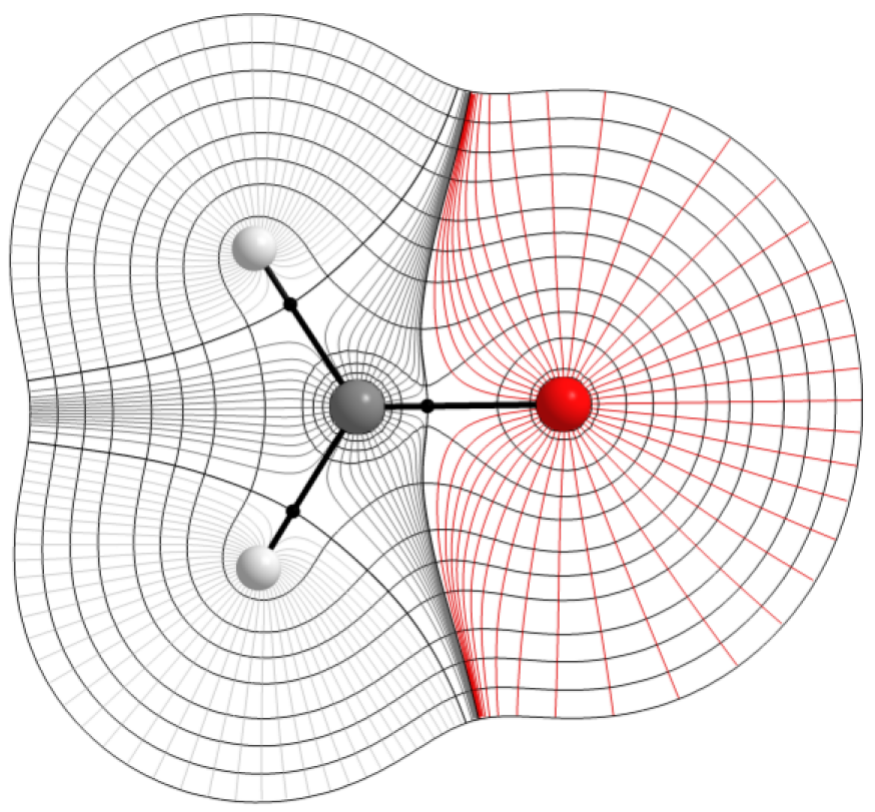
Contour plot of formaldehyde partitioned by gradient paths. Contours connect points with an equal electron density. A collection of gradient paths terminating at the nuclei make up an object called a topological atom. The gray (dark and light) and red lines are gradient paths while black circles are bond critical points. The black curves terminating at the bond critical points are IASs, which act as sharp boundaries between the atoms. Maxima in the electron density practically correspond to the nuclei in the system.

QTAIM falls under the umbrella of quantum chemical topology (QCT),^36^ a group of methods that share the idea of quantum mechanical functions being topologically partitioned by a (gradient) vector field. In QTAIM, a gradient vector field is applied to the electron density of a molecule or group of molecules, which can be obtained theoretically or experimentally. The gradient vector field partitions the electron density, revealing gradient paths, which are trajectories of the steepest ascent, connecting critical points in the electron density. Figure 1 illustrates gradient paths partitioning a formaldehyde monomer, which contains a carbonyl group that features much in this study. An interatomic surface (IAS) is a collection of gradient paths that terminate at a saddle point between nuclei, still called bond critical point although^37^ line critical point is conceptually more neutral. Points on an IAS satisfy eq 2,
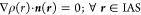
2where ρ is electron density and ***n***(***r***) is a vector normal to the IAS.

In contrast to the fuzzy (i.e., penetrating) atoms generated by various other partitioning schemes,^38−40^ topological atoms are space-filling and do not overlap. Unlike some other methods used in high-resolution crystallography,^41^ QTAIM does not require any parameters or reference densities, with all information coming from the molecular electron density itself.^36^

### 2.2 Energy Definitions: Interacting Quantum Atoms

IQA is an energy decomposition method extending on QTAIM. While QTAIM is only applicable to the energies of atoms in molecules at stationary points on their potential energy surface, IQA is free from this limitation due to the ability^12,42^ to calculate the potential energies of topological atoms without relying on the atomic virial theorem. As shown in eq 3, IQA partitions atomic energies () into intra- () and interatomic () energies rather than separating energies into bonded and nonbonded interactions as done by classical force fields,
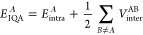
3

The intra- and interatomic energies can be further decomposed, as shown in eqs 4 and 5, where *T* is kinetic energy, *V* is potential energy, and *n* and *e* represent interactions involving nuclei and electrons of atoms *A* and *B*, respectively. Atoms *A* and *B* here should of course not be confused with Buckingham parameters *A* and *B* from eq 1.
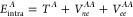
4
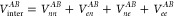
5

Once partitioned using IQA, the deformation energy of a single atom is found as the difference between its intra-atomic self-energy within a system  (here a homomonomeric dimer with carbonyl groups aligned) and the equivalent energy in a “free” system  (here a monomer).
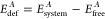
6

Further details of the construction of the dimers studied in this work are given in Section 3.2. The deformation energy between two atoms is then the sum of the deformation energies of the atoms,
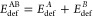
7

This total deformation energy, , is then fitted to an expression that contains the internuclear distance *r*_*AB*_, such as the repulsive term in the Buckingham potential of eq 1.

It is important to pause for a moment in order to appreciate the shift that takes place here in the interpretation of interatomic repulsion away from that offered by the usual classical potential. According to eq 7, this repulsion is now a sum of intra-atomic effects rather than a direct interatomic effect such as the Coulomb potential. However, the appearance of *r*_*AB*_ in the Lennard-Jones potential gives the impression that its repulsive part is a direct interatomic effect. Yet, eq 7 says otherwise. Topological atoms allow a natural and intuitive (even visual) expression of repulsive energy as arising from a mutual and perfectly additive deformation of two interacting atoms. Still, the idea of deformation has featured in the literature for decades, outside of topological atoms, in refs (1, 43), and (44), for example.

In the follow-up work by others mentioned above, the deformation energy is partitioned into a sum of steric (*E*_ST_) and charge transfer energies (*E*_CT_). Steric energy can therefore be calculated as
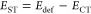
8

The steric energy can also be fitted to an exponential function of the same form as that of the repulsive term in eq 1. A practical solution to quantify the charge transfer energy *E*_CT_ was proposed^31^ by invoking grand canonical density functional theory.^45^ This theory makes one think of the energy of a system, in our case an atom in a dimer, as varying linearly with the number of electrons *N*, even if this number is non-integer. We need to introduce the integer part of *N*, denoted by [*N*], and the fractional part of *N*, denoted as {*N*}. The slope of the line between two consecutive integer numbers of electrons [*N*] and [*N*]+1 is the ionization potential IP, evaluated at [*N*]+1. Note that the named potential is actually an energy, rigorously speaking, as a potential is an energy per charge. However, we keep using the misleading term “potential” for the sake of compatibility with the literature. We also introduce a reference state with electron count *N*^0^, in our case the atom in question in a monomer. Since |*N*−*N*^0^| < 1 in the systems studied here, [*N*] = [*N*_0_]. We retrieved eqs 9 and 10 from refs (46) and (47), respectively,
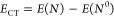
9
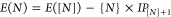
10

Here, *IP*_[*N*]+1_ is the ionization energy going from an electron count of [*N*] to [*N*]+1 where *N* is the number of electrons, or electron population, on an atom in a dimer, and *N*^0^ is the number of electrons on the atom in a monomer. Twice substituting eq 10 into eq 9, once for *N* and once for *N*^0^, leads to
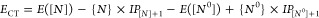
11

Since [*N*] = [*N*_0_], the first and third terms cancel, leaving
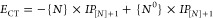
12

Taking the ionization energy out as a factor we obtain
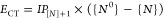
13

**Figure 2:**
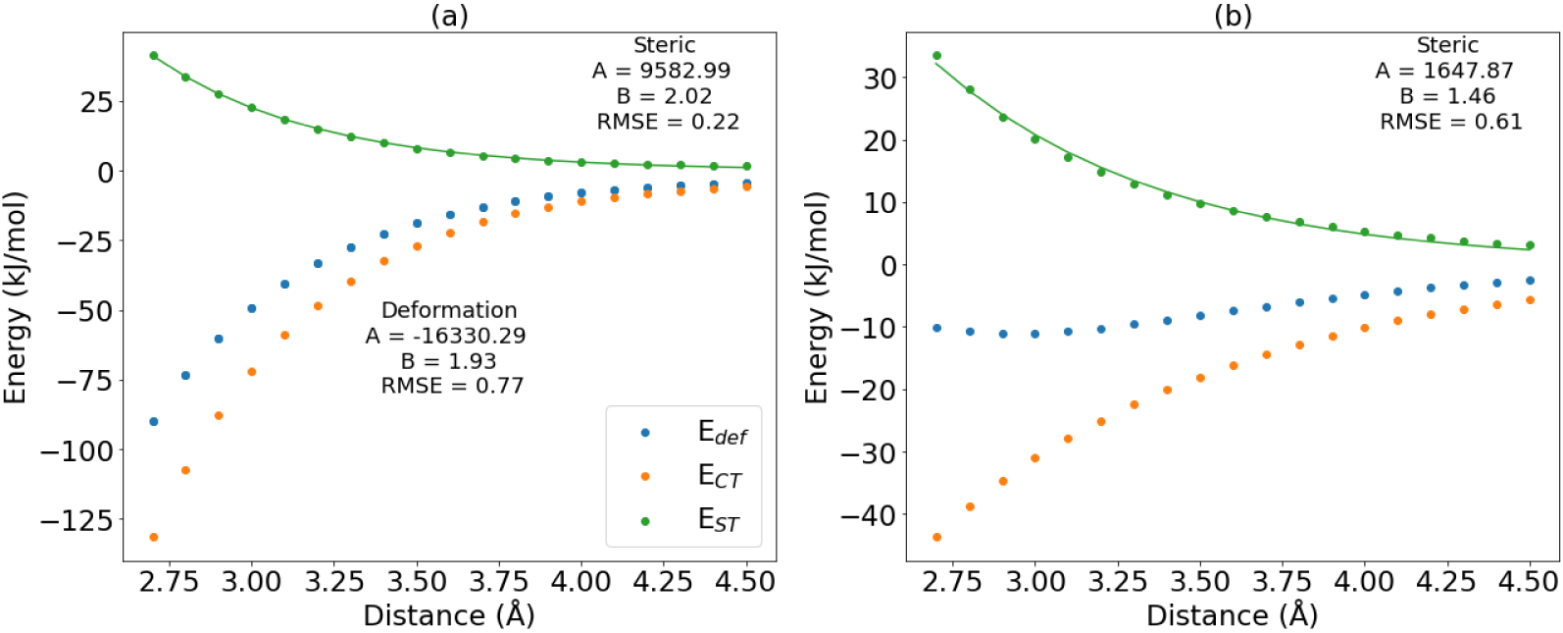
Fitted curve and energy decomposition for carbonyl carbon in (a) carbonic acid (H_2_CO_3_), and in (b) formaldehyde (H_2_CO).

This can be simplified to
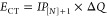
14where Δ*Q* is the difference in electron populations. The removal of the charge transfer term from the deformation energy also corrects problems with carbon atoms that were seen in unpublished work by our group. In some cases, carbon atoms were seen to return a negative deformation energy in the very short range. This is impossible because the corresponding attractive interaction cannot be associated with the desired repulsion. However, application of the charge transfer correction led to energy profiles with the expected repulsive behavior. This effect is illustrated in Figure 2, which shows two case studies: formaldehyde and carbonic acid. Both show negative deformation energies (*E*_def_), which are both fixed by the charge transfer correction (*E*_CT_), leading to an expectedly positive steric energy (*E*_ST_), well fitted to an exponential function. However, we note that the (original, negative) deformation energy does not even show exponential behavior for the case of formaldehyde. This behavior may be linked to polarizability: as the distance decreases, polarization could cause a change in electron density leading to nonexponential behavior.

## 3 Computational Details and Data Set

### 3.1 Data Set

For this work, we selected a wide variety of carbonyls, since the carbonyl group is ubiquitous in chemistry and contains a planar region due to the sp^2^ carbon. This planarity enables molecules to approach each other such that thereby sufficiently exposed carbons atoms can experience each other’s repulsion. This situation makes it possible to readily study patterns in atomic deformation energy between carbon atoms. Amines, amides, and fluorinated molecules were also included to ensure a wider distribution of physical properties such as molecular mass and dipole moment. Table 1 provides a full list of 26 molecules studied after heuristic selection. We note in passing that recent developments in inverse molecular design using generative models (e.g., with polycyclic aromatic systems^48^ may improve this selection, especially if more complex molecules are investigated. There are subsets of the molecules studied here such that the effect of sequential substitution starting from formaldehyde can also be investigated to recover trends predicted by chemical intuition, as discussed later in Section 4.2. For example, we started with formaldehyde and replaced the first one and then both hydrogen atoms with fluorine atoms to examine how attaching electronegative atoms to the carbonyl group affects the parameters *A* and *B*.

**Table 1:**
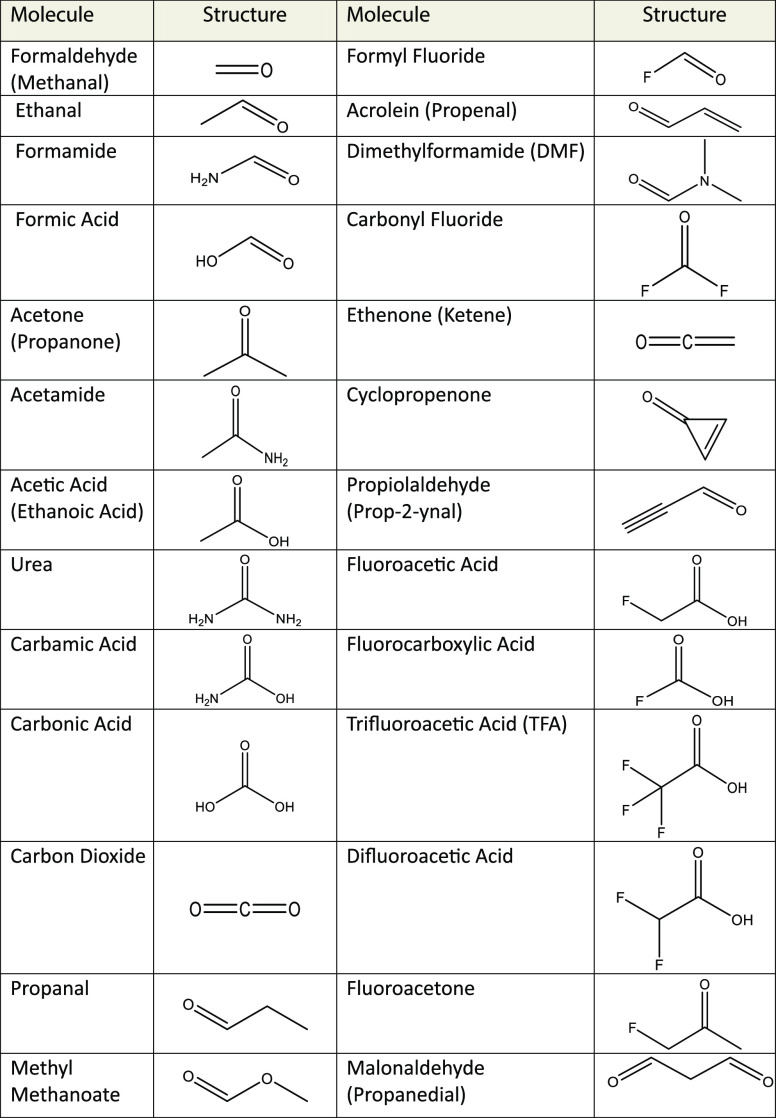
Test Set of 26 Molecules (Systematic IUPAC Name in Parentheses if Common Name Is Given) Used in Scans to Obtain *A* and *B* Buckingham Parameters for Carbonyl Carbon and Oxygen Atoms

### 3.2 Calculation of Complexes (Dimers)

**Figure 3:**
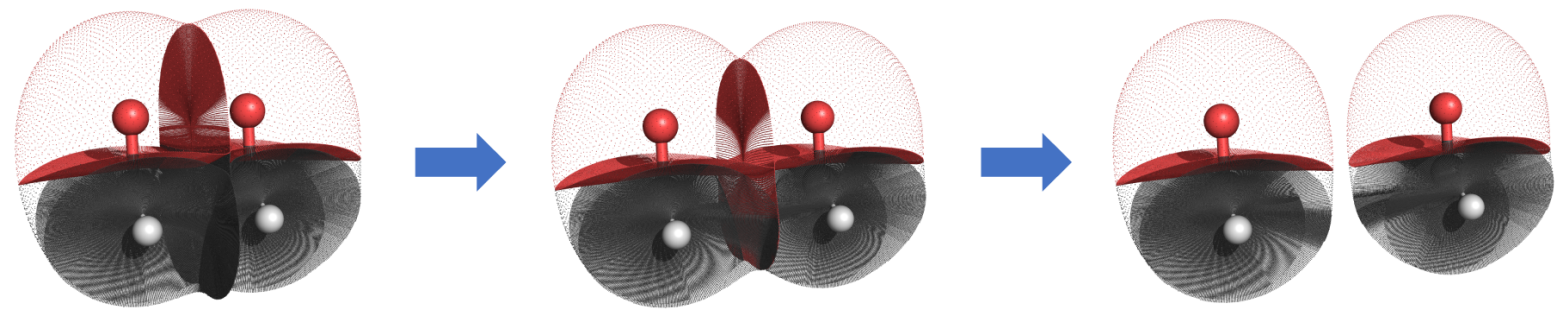
Scan of formaldehyde dimer with carbon and oxygen topological atoms. Note that the surfaces are based on a cutoff for electron density; the topological atoms actually extend to infinity or until there is another atom and are therefore still touching in the final image on the right. These images were prepared with the in-house code PyMol-QTAIM Visualizer, written by Mr F. Falcioni and Dr M.J. Burn.

GAUSSIAN09^49^ was used to optimize monomers of the 26 molecules shown in Table 1 at the B3LYP/aug-cc-pVTZ level of theory. All optimizations were unconstrained except for malonaldehyde, where the two carbonyl groups were kept parallel to mitigate overlap issues from atoms being too close together. The optimized geometries were used to create “face-to-face” dimers where both carbonyl groups were parallel, with intermolecular separations from 2.0 to 4.6 Å in increments of 0.1 Å, as illustrated by Figure 3. Where possible, methyl groups were rotated to maximize the total distance between corresponding hydrogen atoms of the dimers. In other words, this rotation was performed to allow for smaller intermolecular separations before hydrogen atom overlap caused the atomic property program AIMAll to fail. Monomers were aligned and moved along an axis perpendicular to the plane of the carbonyl region and centered on the carbon atom of one of the monomers. Intermolecular separation can then be increased thereby ensuring a fair test, i.e., avoid anomalies due to angular dependence and substituents. The scan range for each atom type is tabulated in Supporting Information Section 1, that is, in Table S1.

For each of the 26 face-to-face dimers, at each separation, a single-point calculation was performed using GAUSSIAN09 to compute the dimeric wavefunctions. The wavefunctions of the two constituent monomers (each with identical internal geometry) that make up each dimer were also calculated. The IQA partitioning was then implemented using the QTAIM program^50^ AIMAll (version 19) to partition the dimers and monomers into topological atoms with a basin outer angular quadrature of gs30, using the Proaim integration method. Intra-atomic energies and atomic charges for carbon and oxygen atoms were then extracted, enabling the deformation and charge transfer energies to be found as described earlier in Section 2.2. The chosen scan ranges avoided AIMAll calculations failing due to methylene hydrogen atom overlap. The deformation energies between pairs of atoms were then calculated using eqs 6 and 7.

For steric energies to be calculated, a charge transfer term must be calculated using an ionization energy. These calculations were performed in GAUSSIAN09 where the energies of single atoms (and ions with the required charge and multiplicities) were calculated at the B3LYP/aug-cc-pVTZ level of theory. The difference in energies between the two atoms or ions was then used as the ionization energy. The atomic energies are listed in Table S4.

### 3.3 Fitting of the Repulsive Buckingham Potential

The effect of narrowing the range of the scans performed here was also explored (Supporting Information Section 1). Changing the range resulted in different *A* and *B* values and hence different root-mean-square errors (RMSEs) for the fits, given in Tables S2 and S3. Narrowing the range allowed for more accurate fits in most cases but ultimately resulted in diminishing returns in terms of RMSE reductions. Hence, we decided on the widest possible ranges while avoiding errors due to atom overlap, as listed in Table S1. No such issues were seen with nitrogen atoms, which are further explored in Supporting Information Section 2. Hydrogen atoms were not analyzed because they do not contribute significantly to steric interactions,^30^ and the fluorine atoms present in several of the molecules studied were not investigated here because the small intermolecular separations required to study their short-range repulsion would have resulted in problems due to overlap of other atoms. Fluorine has previously been studied in diatomic molecules,^26,30^ where this was not an issue.

The deformation energy change with the internuclear distance was calculated between pairs of carbonyl carbon and oxygen atoms and fitted to the repulsive part of a Buckingham potential (see eq 1). While the full Buckingham potential contains an attractive *r*^–6^ term, it has been excluded here because it is not relevant to the repulsive interactions studied in this work. This procedure was repeated for the steric energy by using eqs 8 and 9.

Relying on non-linear least-squares regression, the fitting was performed using the Levenberg–Marquardt algorithm,^51^ which finds the nearest local minimum.^52^ This local minimum is not necessarily the global minimum, which can become a problem if the initial guess provided for the parameters is too far from the global minimum. To make sure the initial guess used in the fits performed in this work was suitable, the values of the initial guesses were varied, and the potential compared to the data by RMSE. Upon changing the initial parameter guesses from 10^3^ to 10^5^ kJ mol^–1^ in steps of 1,000 kJ mol^–1^ for *A*, and 1 to 10 in steps of 0.1 Å^–1^ for *B*, we found no change in the final parameters. Therefore, our values for *A* and *B* should be stable and unique. For molecules with chemically equivalent atoms, namely, the oxygens in carbon dioxide and both carbonyl atoms in malonaldehyde, only one was arbitrarily selected for analysis with the assumption that the other atom(s) would behave identically.

## 4 Results and Discussion

### 4.1 Transferability of the *A* and *B* Repulsive Buckingham Parameters

**Figure 4:**
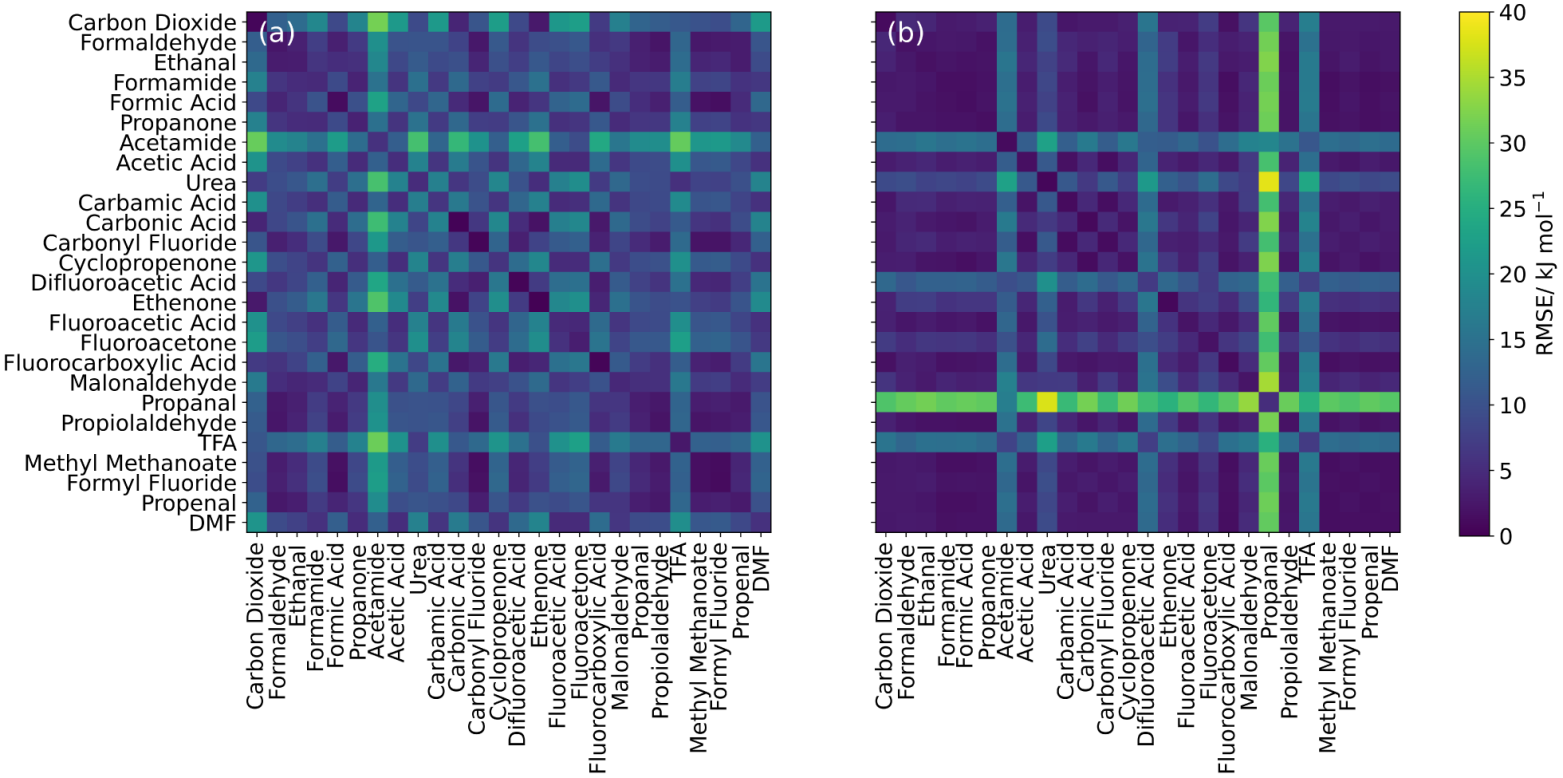
Heatmaps showing the transferability of *A* and *B* parameters of the carbonyl oxygen atom in all 26 molecules for (a) the deformation energy and (b) the steric energy. Diagonal cells represent the RMSE of the fitted Buckingham potential and the data to which it was fitted, while off-diagonal cells represent transferability RMSEs of the Buckingham curve plotted using the *A* and *B* values of the molecule in the row compared to the data of the molecule in the column.

**Figure 5:**
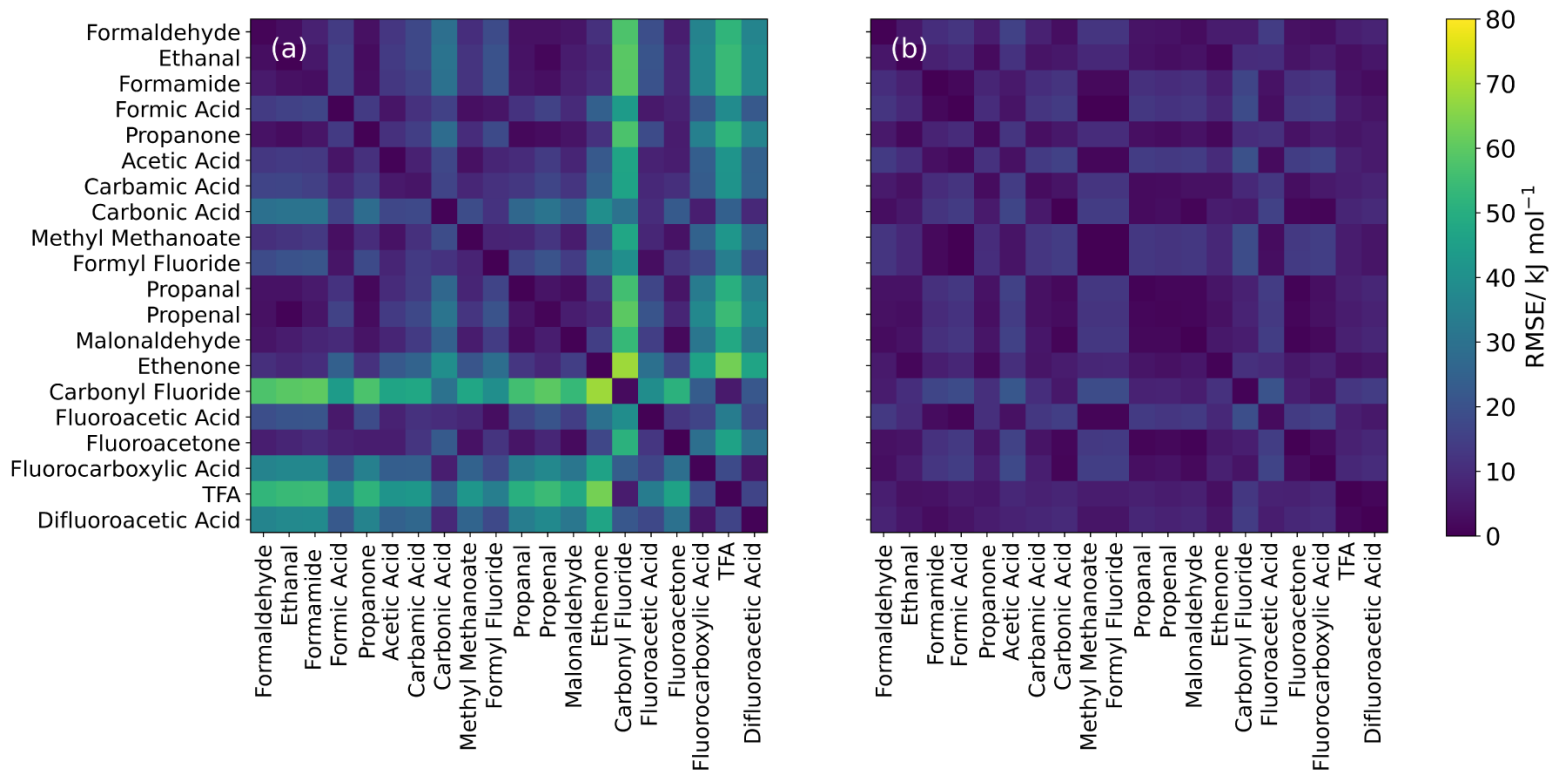
Heatmaps showing the transferability of *A* and *B* parameters of the carbonyl carbon atom in 20 (instead of 26) molecules for (a) deformation energy and (b) steric energy. Diagonal cells represent the RMSE of the fitted Buckingham potential and the data it was fitted to, while off-diagonal cells represent transferability RMSEs of the Buckingham curve plotted using the *A* and *B* values of the molecule in the row compared to the data of the molecule in the column.

The quality of fits to the deformation and steric energies was measured by RMSE, with a lower value indicating that the fitted potential better represented the collected data. The steric and deformation energy data could be fitted with RMSEs of less than 7.0 kJ mol^–1^ for oxygen and 11.6 kJ mol^–1^ for carbon. These fits are represented by the cells along the main diagonals of the heatmaps in Figure 4 for the carbonyl oxygen and in Figure 5 for the carbonyl carbon. Some carbon deformation data could not be fitted to an exponential function due to the problems mentioned in Section 2.2 and depicted in Figure 2. Out of a total of 98 fits, 83 of the fitted RMSEs (approximately 85%) were smaller than 4 kJ mol^–1^. Values for *A* and *B* and their associated RMSEs are listed in Supporting Information Section 3.

As well as indicating the quality of the fits, Figures 4 and 5 also show the transferability of the Buckingham parameters in the off-diagonal cells of the heatmaps. Transferability was quantified by the RMSE between the Buckingham potential with fitted parameters *A* and *B* for an atom in one molecule *X* and the original, exact deformation (or steric) energy data of the same atom type in another molecule *Y*. This calculation is expressed in eq 15,
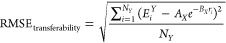
15where *A*_*X*_ and *B*_*X*_ are the fitted Buckingham parameters for molecule *X*, *N*_*Y*_ is the number of data points for molecule *Y*, *E*^*Y*^ is the deformation (or steric) energy for molecule *Y*, and *r*_*i*_ is the intermolecular separation at the *i*-th data point of molecule *Y*.

We consider the potential to be transferable if the RMSE value is smaller than 4 kJ mol^–1^. This roughly equates to 1 kcal mol^–1^, the widely accepted limit for chemical accuracy.^53^ This approach was chosen over directly comparing *A* and *B* values because it better represents how the parameters capture the shape of the deformation and steric energy curves, which would be relevant if transferability was being used in a force field.

Table 2 lists the average, minimum, and maximum RMSEs from the transferability heatmaps. It turns out that not all carbon deformation data could be fitted to an exponential curve, which affected 6 out of a total of 26 molecules. For this reason, there are two rows in Table 2 for carbon steric energy: one showing summary statistics calculated over all 26 molecules, and one showing summary statistics calculated for only the 20 molecules for which deformation energy showed exponential behavior. This arrangement allows for a fair comparison of deformation and steric transferability for carbon atoms. For the same reason, Figure 5 only contains 20 of the 26 molecules. A heatmap showing transferability of *A* and *B* for steric energy of carbon atoms in all 26 molecules can be found in Supporting Information Section 3 (Figure S3b).

**Table 2:** Minimum, Maximum and Average RMSE (in kJ mol^–1^) of the Transferability RMSEs for Deformation and Steric Energies of Carbonyl Carbon and Carbonyl Oxygen Atoms

atom		average	minimum	maximum	% < 4 kJ mol^–1^
O	deformation	10.3	1.4	31.6	11.2
	steric	8.2	1.3	38.4	44.8
C	deformation	21.5	0.8	68.4	8.2
	steric (in deformation)	7.8	0.2	21.6	27.6
	steric (all)	8.8	0.2	29.2	26.3

Figures 4a and 5a show that the transferability of Buckingham parameters from fits to IQA deformation energies is generally better for oxygen atoms. Indeed, a comparing glance reveals more entries (i.e., little squares) with higher RMSE values in Figure 5a, which reports on the carbons. This conclusion is corroborated with the corresponding numbers in Table 2, showing an average RMSE of 10.3 kJ mol^–1^ for oxygen, which is lower than that for the carbons (21.5 kJ mol^–1^). The least transferable potentials (highest RMSE values) tend to be between molecules with significantly different structures. For example, plotting the parameters from the fitting of carbonyl fluoride (a carbonyl group with two fluorine atoms attached to carbon) with the data from ethenone (a ketene) gives the largest error of 68.4 kJ mol^–1^ for the carbon deformation energy. On the other hand, molecules with similar chemical environments such as ethanal and propanal (both aldehydes, without perturbing substituents) have good transferability in deformation energy with an RMSE of 3.9 kJ mol^–1^ for carbon and 3.7 kJ mol^–1^ for oxygen. This heightened transferability between atoms in similar chemical environments suggests that specific atom types should be defined based on chemical environment, accounting for substituents, instead of only considering the functional group that the atom is part of. Indeed, this is the approach taken by classical force fields such as AMBER^54^ which, as an example, differentiates between sp^3^ carbons in three- and four-membered rings.

The poorer performance of carbon in Figure 5 compared with oxygen in Figure 4 may be due to charge transfer effects. That is, the carbonyl carbons studied are often bonded to heavy (non-hydrogen) atoms that are more likely to cause charge transfer within the system than the singular carbon atom bonded to the oxygen atoms. The improvement seen in the transferability on going from deformation energy to steric energy, where an approximation to the charge transfer has been removed (Figures 4b and 5b), further backs up this argument. However, it is possible that the correction does not fully remove charge transfer effects, since in some cases applying the correction results in a negative steric energy (discussed further in Supporting Information Section 3).

The parameters obtained from fitting the steric energy almost always resulted in lower transferability RMSEs (indicating better transferability), with the average transferability RMSE decreasing by 12.3 kJ mol^–1^ (57%) and 2.1 kJ mol^–1^ (21%) for carbon and oxygen, respectively. In the few cases where the steric correction led to a higher RMSE than the deformation fit, the issue was likely integration error or the approximation for charge transfer. The proposed^32^ charge transfer correction uses the floor function to take the integer number of electrons. However, in calculations performed in this work, rounding the charge the other way (by using a ceiling function), or even using the next ionization energy sometimes gave a better fit. Examples where rounding differently leads to a better fit are shown in Supporting Information Section 3 (Table S9). Across the data sets for both carbon and oxygen atoms, 36% of steric energy errors were below 4 kJ mol^–1^, while only 10% of deformation energies were able to meet this criterion for transferability.

Transferability of the *A* and *B* parameters is significantly worse for nitrogen, with some off-diagonal RMSEs of over 100 kJ mol^–1^. Plots for nitrogen can be found in (Supporting Information Section 2, Figures S1 and S2). Poor transferability is likely a result of not having enough data to differentiate between different functional groups involving nitrogen. That is, transferability could again be greatly improved by defining more specific atom types such as amines connected to aliphatic chains and amines connected to aromatic rings. Future work on a larger data set could investigate the feasibility of this approach with IQA data.

The lack of transferability of some outliers for nitrogen, as well as for carbon and oxygen, can be rationalized in terms of moieties that are present in the molecule but not in other molecules studied. For example, even after narrowing the scan range to avoid atom overlap, the methylene group of propanal still spoils the transferability. Looking at further examples, cyclopropenone and nitroxyl, which show poor transferability, are the only molecules in the data set with a ring and N=O bond, respectively. Interestingly, the outliers are not consistent: outliers in the carbon transferability plot are not necessarily outliers in the oxygen or nitrogen transferability plots. Therefore, transferability is dependent on chemical environment and atomic identity rather than either factor individually or on some property of the whole molecule.

### 4.2 Physical Meaning of *A* and *B* Parameters

**Figure 6:**
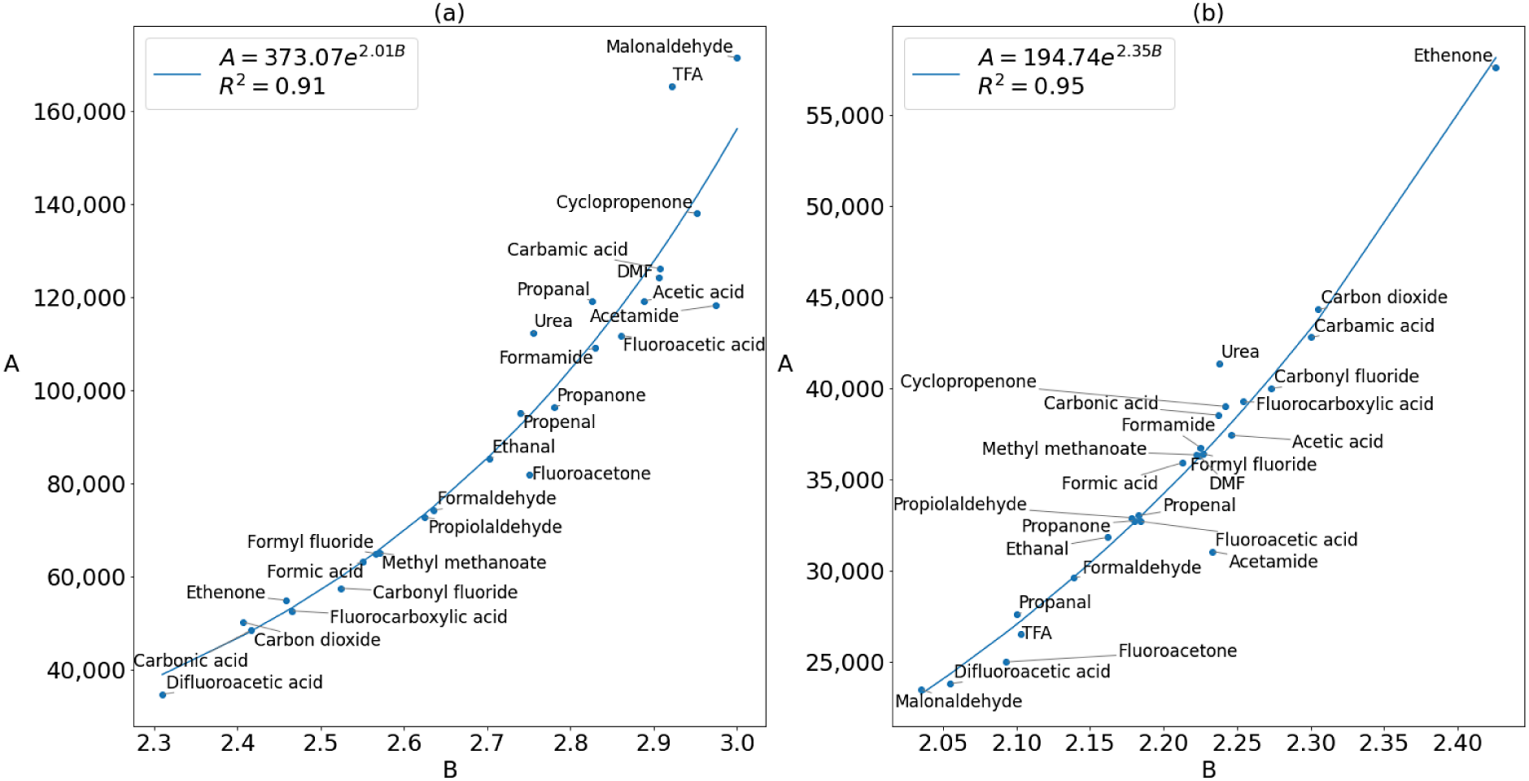
Plots of the fitted *A* against fitted *B* values for the oxygen (a) deformation and (b) steric energy.

**Figure 7:**
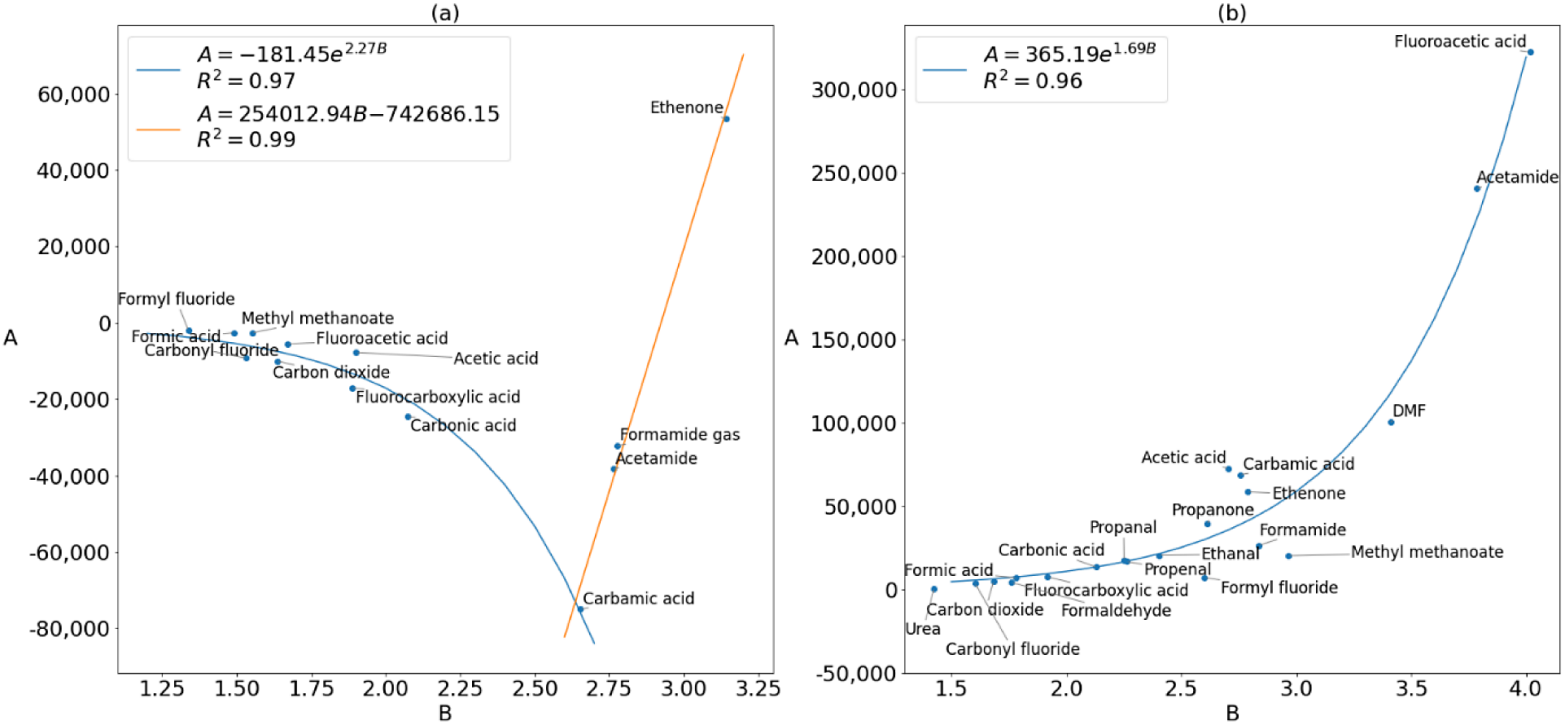
Plots of the fitted *A* against fitted *B* values for the carbon (a) deformation and (b) steric energy.

The concept of transferable parameters implies that the parameters have a physical meaning. During this work, several strong correlations were observed between the derived parameters, which may form the basis of future investigation. The strongest of these relationships was between the *A* and *B* parameters themselves, with *R*^2^ values above 0.9 as shown for oxygen in Figure 6 and for carbon in Figure 7.

Exponential relationships between *A* and *B* are also present but not commented upon in the data^26^ of Wilson and Popelier (Figure S4 in Supporting Information Section 4). At first, this would appear to be a mathematical artifact as there may not necessarily be any physical relationship between the parameters; few physical properties are correlated at all,^55^ and we are not currently aware of any properties that are exponentially correlated. Nevertheless, relationships between Buckingham parameters have been seen before with short-range repulsion from DFT studies.^56,57^ These relationships suggest that it may be possible to fit Buckingham curves for short-range repulsion by using only one parameter. This can be done at least over the distance ranges used in this work, or possibly for a given atom type because *A* can be written in terms of *B* with small errors. In general, however, *A* and *B* cannot be redundant: an exponential still needs to be defined by two parameters. Indeed, a logarithmic plot of an exponential function is a straight line, which is fully defined only by gradient *B* and intercept *A*.

In Figure 7a, the sudden transition from an exponential curve to a straight line suggests that the relationship between deformation parameters *A* and *B* is influenced by charge transfer effects because this transition is not present in the steric *A* versus *B* plot. Therefore, this transition has a physical basis. If such a transition and physically grounded relationship between *A* and *B* exist for carbon, then the exponential relationships for the oxygen and nitrogen could also have a physical meaning behind them. For nitrogen, which is the atom with the widest scan range in this study and also the worst transferability, the deformation *A* and *B*, plotted in Figure S2, show a weaker exponential relationship with an *R*^2^ value of 0.86, while the steric parameters show no relationship. Perhaps the *R*^2^ value of the plot of *A* against *B* can be used as an indicator of transferability within a given set of molecules, since the atoms with good transferability also have high *R*^2^ values for their *A* versus *B* plots. Future work could include studying simpler systems in greater detail over a wider range of distances to further elucidate the relationship between *A* and *B* within IQA.

Looking at the *A* and *B* values themselves, we also considered whether trends matching chemical intuition might exist when changing the degree of substitution, as with the trends in nucleophilicity and electrophilicity recovered before.^32^ Starting with formaldehyde, we can substitute one or both of the hydrogen atoms with another atom or group; the three hydrogen atoms on a methyl group such as the one in acetic acid can all be substituted as well. As an example, Table 3 shows how going from formaldehyde over formyl fluoride to carbonyl fluoride, the oxygen deformation *A* and *B* decrease, in line with the chemical intuition of fluorine inductively pulling electron density away from the oxygen atoms, leading to less repulsion. The same trend is seen for the sequence formaldehyde/formic acid/carbonic acid, where hydrogens are systematically replaced by OH groups. Interestingly, the trend is reversed after the steric correction, suggesting that electron-withdrawing groups increase the steric hindrance experienced by the oxygen atom.

**Table 3:** Trends in Carbonyl Oxygen Parameters *A* and *B* with Increasing Substitution (F or OH)

molecule	deformation *A*/kJ mol^–1^	deformation *B/*Å^–1^	steric *A*/kJ mol^–1^	steric *B*/Å^–1^
formaldehyde	96,444.3	2.74	23,708.2	2.05
formyl fluoride	72,760.7	2.61	30,265.0	2.16
carbonyl fluoride	60,352.5	2.54	34,299.7	2.21
formic acid	71,351.6	2.60	30,329.9	2.15
carbonic acid	48,924.7	2.42	34,390.2	2.19

Plots of deformation *A* and *B* parameters (Figure S5 for oxygen and Figure S6 for carbon) against the molecular dipole moment have weak correlations, which are not present after applying the steric correction. This effect occurs because a dipole moment is related to atomic charges, which are subtracted from deformation energy when applying the steric correction. We then plotted (in Figure S7) steric *A* and *B* against topological atom volume, expecting a relationship between steric energy and through-space compression of atomic volumes because steric effects are classically linked to the size and shape of molecules. However, no such relationship was found at any intermolecular separation, raising the question of which physical properties the steric *A* and *B* may be linked to.

## 5 Conclusions

This work studied short-range repulsion of carbon and oxygen atoms in dimers of carbonyl compounds consisting of monomers containing up to 12 atoms. We have increased our understanding of the repulsive Buckingham parameters for deformation energy, which represents short-range repulsion, between pairs of identical atoms and applied a previously proposed charge transfer correction to do the same for steric energy. Hence, we have shown that these parameters are useful and meaningful rather than mathematical trinkets.

This study has shown that this charge transfer correction is especially important for carbon–carbon short-range interactions. Without the correction, these interactions can display nonexponential behavior and, in some regions, even appear attractive. Applying this correction is shown to recover the expected exponential behavior with few exceptions, indicating that charge transfer was indeed responsible for the problems we saw with carbon in previous work.

We have learned that the parameters obtained from the Buckingham fit are transferable. This transferability is proven for carbonyl carbon and oxygen atoms in a variety of molecules with an average error of 21.5 and 10.3 kJ mol^–1^ for the deformation energies of carbon and oxygen, respectively. The charge transfer correction generally improves this further, with the average error decreasing to 8.8 and 8.2 kJ mol^–1^ again for carbon and oxygen, respectively. The larger improvement seen with carbon is ascribed to a greater contribution of charge transfer effects due to the additional groups bonded to the carbon relative to the oxygen, which is only bonded to one atom. Without discriminating between the different chemical environments around the carbonyl group, across the data sets for both carbon and oxygen atoms 36% of the steric transferability errors and 10% of the deformation transferability errors are below 4 kJ mol^–1^, and thus within the often quoted 1 kcal mol^–1^ chemical accuracy. In some cases, the parameters are transferable even without the correction, such as between formaldehyde and ethanal. In other words, the correction improves not only the quality of fits but also transferability.

Finally, plotting parameter *A* directly against *B* reveals that these two parameters are closely related to each other in a way that is suggestive of physical meaning. These relationships potentially offer a way to describe short-range interactions for an atom type by fitting only one parameter as well as another way to quantify transferability and define or verify atom types in the first place. This phenomenon does not seem to have been noticed or exploited in previous work in this area.

## Data Availability

The data supporting the findings reported in this paper are openly available from the *Data for: Transferability of Buckingham Parameters for Short-Range Repulsion between Topological Atoms* repository at DOI: 10.17632/pt2rw9rh6j.1

## Supporting Information Available

Section 1: Scan ranges (Table S1); minimum, maximum and average RMSEs when changing scan range (Tables S2 and S3); atomic energies used to calculate ionization energies (Table S4). Section 2: nitrogen transferability heatmap (Figure S1) and plot of *A* against *B* (Figure S2). Section 3: fitted parameters from different scan ranges (Tables S5–8); RMSE when rounding differently while approximating charge transfer (Table S9); molecules where charge transfer correction results in negative steric energy (Table S10); carbon transferability heatmap for all molecules (Figure S3). Section 4: plot of *A* against *B* with data from Wilson and Popelier (Figure S4); plots of oxygen and carbon steric *A* and *B* values against molecular dipole moment (Figures S5-6); plots of carbon *A* and *B* values against topological atom volume (Figure S7) (PDF)
