# Supplementary material for: Transferability of Buckingham Parameters for Short-Range Repulsion between Topological Atoms
Source: J Phys Chem A. 2024 May 28;128(22):4561–72. doi: 10.1021/acs.jpca.4c02048 (PMC11163427; doi:10.1021/acs.jpca.4c02048)
Supplement: Supplementary file 1 — jp4c02048_si_001.pdf [file jp4c02048_si_001.pdf]

Supporting Information

Transferability of Buckingham Parameters for Short-range Repulsion between Topological Atoms

Jaiming J. K. Chung, Matthew L. Brown and Paul L. A. Popelier*

Department of Chemistry, The University of Manchester, Oxford Road,
Manchester, M13 9PL, United Kingdom

Phone: +44 161 3064511. Email: pla@manchester.ac.uk

Contents

1	Additional Computational Details	S2
2	Nitrogen	S4
3	Fitted Parameters	S5
4	Properties	S11
5	References	S13

1. Additional Computational Details

The scan range is the range of interatomic distances over which the deformation and steric energies were studied. Wilson and Popelier used a range of 70% to 130% of the sum of van der Waals radii of the two atoms involved[1]. In this work, we used ranges starting at larger interatomic distances and ending at the same distance as Wilson's range. This was done to reduce the effect of hydrogen atom overlap in the dimers as described in the main text.

Table S1. Scan ranges used for deformation and steric energy calculations.

Atoms	Previous Range[1] / Å	Range in this work / Å
C-C	2.3 — 4.5	2.7 — 4.5
O-O	2.1 — 4.0	2.3 — 4.0
N-N	2.1 — 4.1	2.1 — 4.1

Moreover, we noticed a secondary benefit to using a narrower scan range: scans performed using narrower ranges give lower values for the fitted average, minimum and maximum RMSE, as shown in Tables S2 and S3. For each deformation energy plot, data points were removed starting from the smallest distance because removing data points from the furthest distance would lead to little or no change in fitting due to the exponential shape of the repulsive Buckingham curve. The remaining data points were then fitted to the same Buckingham potential, eq S1, giving new values of A and B.

$$E(r) = Ae^{-Br} \qquad (S1)$$

Table S2. Minimum, maximum and average **oxygen** deformation RMSE (in kJ mol^{-1}) when changing the scan range (excluding propanal and malonaldehyde due to hydrogen overlap errors < 2.3 Å).

Range / Å		2.1-4.0	2.2-4.0	2.3-4.0	2.4-4.0	2.5-4.0
Deformation	Average	3.9	3.2	3.0	2.8	2.6
	Minimum	0.4	0.3	0.3	0.3	0.1
	Maximum	13.0	6.4	6.1	5.9	5.7
Steric	Average	3.0	2.2	2.0	1.6	1.1
	Minimum	0.8	0.8	0.7	0.6	0.6
	Maximum	12.7	6.9	7.0	6.8	1.6

Table S3. Minimum, maximum and average **carbon** deformation RMSE (in kJ mol^{-1}) when changing the scan range. "In deformation" refers to the subset of molecules where deformation energy displayed exponential behaviour.

Range / Å		2.3-4.5	2.4-4.5	2.5-4.5	2.6-4.5	2.7-4.5	2.8-4.5
Deformation	Average	4.2	3.6	1.4	1.2	1.0	0.9
	Minimum	0.4	0.3	0.2	0.2	0.2	0.2
	Maximum	25.8	26.4	4.7	4.6	4.5	4.3
Steric (in deformation)	Average	1.6	1.4	0.9	0.9	0.8	0.8
	Minimum	0.1	0.1	0.1	0.1	0.0	0.0
	Maximum	8.0	6.3	3.4	3.4	3.5	3.6
Steric	Average	3.1	1.8	1.4	1.3	1.3	0.9
	Minimum	0.1	0.1	0.1	0.1	0.0	0.0
	Maximum	39.6	11.3	11.1	11.3	11.6	3.6

Although progressively narrowing the range further improves average RMSEs, it also diminishes the exponential behaviour of the data. Therefore, we chose a scan range starting from 2.3 Å for oxygen, and from 2.7 Å for carbon, in order to reduce the effect of non-exponential behaviour at small distances on the fits while preserving the exponential shape of the data, which would be lost if the truncation were taken too far. Indeed, narrowing the scan range reduces non-exponential behaviour (from errors at small distances). Narrowing the scan range by too much eliminates exponential behaviour (because the data will almost lie on a straight line).

Using eq 14 in the main text (derived from equations first formulated by Costales *et al.* [2]) we calculated the energetic contribution from charge transfer, E_{CT}, by rewriting eq 14 as

$$E_{CT} = \mathrm{IP}_{\lfloor N \rfloor + 1} \times (\mathrm{N} - \mathrm{N}^0) \quad \text{(S1.2)}$$

where N is the electron count of an atom in a system (here a dimer), N^0 is the electron count of an atom in a reference state (here a monomer), and the ionisation potential IP is the difference in energy between an atom with a charge of $\lceil N \rceil$ (ceiling of noninteger $N, \mathrm{e.g.}\ 2 \text{ if } \mathrm{N} = 1.6$), and that same atom with a charge of $\lfloor N \rfloor$ (floor of noninteger N, e.g. 1 if $N = 1.6$). For example, the ionisation potential (more rigorously called energy) of a carbon atom with a charge between +1 and +2 would be calculated as $\mathrm{IP_C} = \mathrm{E}(\mathrm{C}^{2+}) - \mathrm{E}(\mathrm{C}^{+})$. The energies of the atoms and ions used to find these differences are given in Table S4 and were calculated in GAUSSIAN09[3] at the B3LYP/aug-cc-pVTZ level of theory.

Table S4. Atomic energies calculated at the B3LYP/aug-cc-pVTZ level of theory taking into account spin multiplicities.

Atom/Ion	Energy / kJ mol^{-1}
C	-99,399.0
C^+	-98,285.5
C^{2+}	-95,872.0
C^{3+}	-91,314.5
O	-197,159.8
O^-	-197,321.6
O^{2-}	-196,693.2
N	-143,359.9
N^-	-143,380.6
N^{2-}	-142,765.4

2. Nitrogen

While the main text focused on carbonyl carbon and oxygen atoms, data regarding nitrogen atoms are presented in this section. As with carbon and oxygen, the nitrogen (appearing in only 10 out of 26 molecules) deformation and steric energies were fitted to a repulsive Buckingham potential of the form given in eq S1, to yield the parameters A and B. The plots in this section were made in the same way as Figures 4 and 5 in Section 4 of the main text. Transferability is shown in Figure S1, while Figure S2 plots the fitted A values against the fitted B values. Compared to carbon and oxygen, transferability of nitrogen is significantly worse, with no transferability RMSE values smaller than 4 kJ mol^{-1} in Figure S1. Moreover, the exponential relationship between the nitrogen deformation A and B parameters is weaker than the corresponding relationships for carbon and oxygen shown in Figures 6 and 7 in the main text, and absent for nitrogen steric A and B.

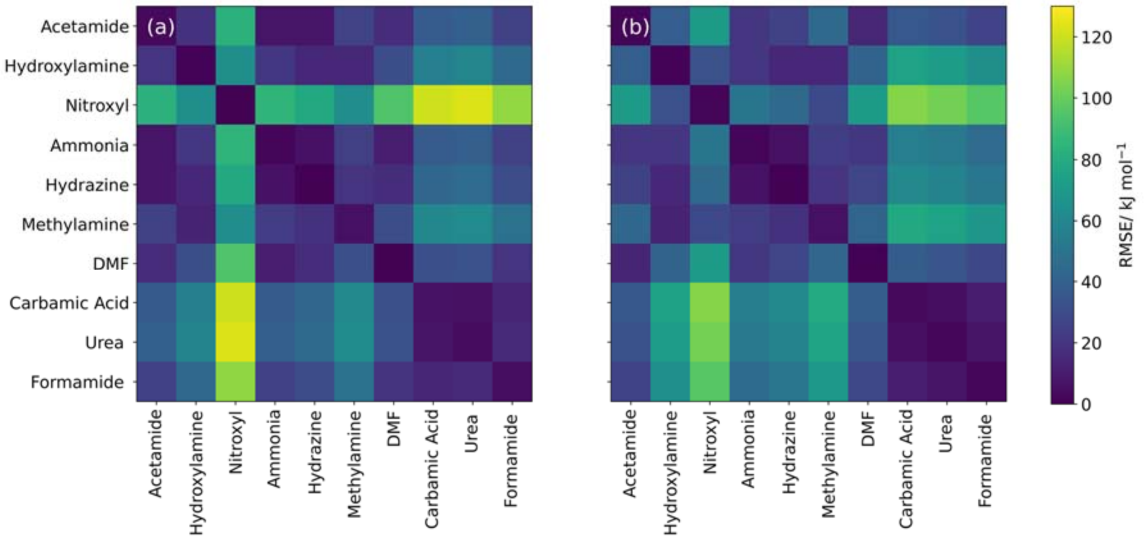

Figure S1. Heatmaps showing the transferability of A and B parameters of the nitrogen atom in 10 molecules for (a) deformation energy and (b) steric energy. Diagonal cells represent the RMSE between the fitted Buckingham potential and the original data it was fitted to, while off-diagonal cells represent transferability RMSEs of the Buckingham curve plotted using the A and B values of the molecule in the row compared to the data of the molecule in the column.

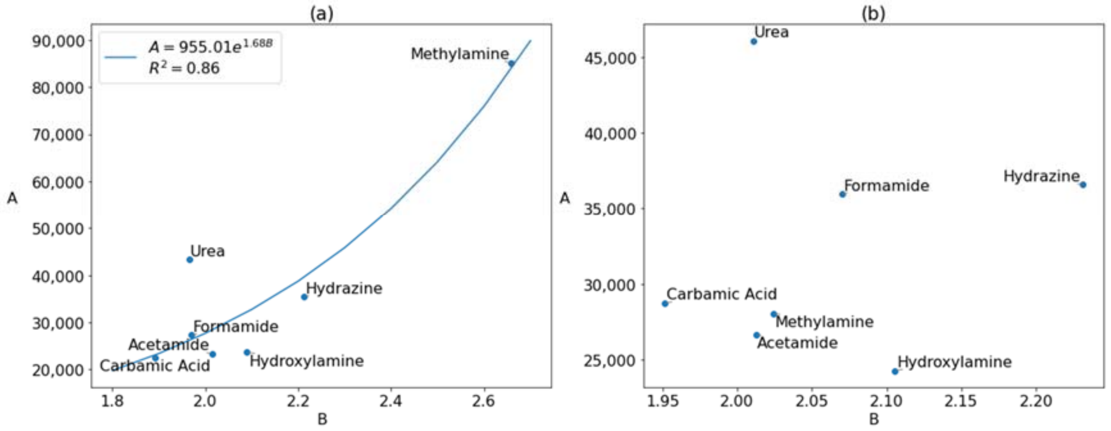

Figure S2 Relationship between the (a) deformation and (b) steric energy A and B parameters for nitrogen atoms.

3. Fitted Parameters

Tables S5 to S8 list the parameters obtained by fitting deformation (“Def”) and steric (“Str”) energy data to a repulsive Buckingham potential with the form of eq S1. Two scan ranges are shown: (i) the original, wider range used by Wilson and Popelier (Table S6 for oxygen and Table S8 for carbon), and (ii) the narrower range used in the main text (Table S5 for oxygen and Table S7 for carbon).

Table S5. Values of the A and B parameters along with the RMSEs of their fits to the deformation and steric energy data for the oxygen atom. The A and B values presented are from the 2.3-4.0 Å scan. Molecular dipole moments, $|\boldsymbol{\mu}|$, are also given.

Molecule	Def A / kJ mol^{-1}	Def B / Å^{-1}	Def RMSE / kJ mol^{-1}	Str A / kJ mol^{-1}	Str B / Å^{-1}	Str RMSE / kJ mol^{-1}	$|\boldsymbol{\mu}|$ / D
Carbon Dioxide	45949.0	2.37	0.6	41505.8	2.28	1.2	0.00
Formaldehyde	96444.3	2.74	2.4	23708.2	2.05	2.0	2.39
Ethanal	121278.4	2.84	3.3	26361.1	2.09	1.8	2.85
Formamide	184653.1	3.04	4.8	30874.6	2.16	1.7	3.95
Formic Acid	71351.6	2.60	1.3	30329.9	2.15	1.5	1.55
Propanone	146698.8	2.95	3.8	27223.0	2.11	1.7	3.00
Acetamide	260070.7	3.29	5.7	27035.4	2.18	1.1	3.91
Acetic Acid	216586.6	3.12	5.2	31568.5	2.18	1.6	4.37
Urea	146788.9	2.86	5.0	40174.4	2.23	0.7	4.35
Carbamic Acid	247554.7	3.17	6.1	37741.3	2.25	1.2	5.20
Carbonic Acid	48924.7	2.42	0.4	34390.2	2.19	1.0	0.31
Carbonyl Fluoride	60352.5	2.54	0.6	34299.7	2.21	1.4	1.03
Cyclopropenone	267325.9	3.21	6.0	31264.0	2.16	2.0	4.33
Difluoroacetic Acid	34714.6	2.31	0.6	9788.8	1.75	6.8	2.85
Ethenone	56551.2	2.47	0.3	54010.2	2.40	0.9	1.52
Fluoroacetic Acid	194726.5	3.08	4.8	26368.4	2.10	2.1	3.88
Fluoroacetone	124278.1	2.91	3.4	20053.2	2.01	2.0	4.20
Fluorocarboxylic Acid	54771.9	2.48	0.6	34532.1	2.20	1.2	2.25
Malonaldehyde	171521.8	3.00	4.7	23477.3	2.04	2.3	4.84
Propanal	119293.5	2.83	3.4	216401.6	3.11	5.2	2.78
Propiolaldehyde	90443.7	2.71	2.2	26863.0	2.10	1.9	3.00
TFA	325679.2	3.17	2.6	9548.5	1.75	7.0	2.82
Methyl Methanoate	75479.8	2.63	1.6	30803.1	2.16	1.5	1.93
Formyl Fluoride	72760.7	2.61	1.2	30265.0	2.16	1.6	2.11
Propenal	112291.7	2.80	2.9	30244.9	2.15	1.5	2.82
DMF	236822.2	3.16	5.7	30484.4	2.16	1.6	4.21

Table S6. Values for the A and B parameters along with the RMSEs of their fits to the deformation and steric energy data for the oxygen atom in the 2.1-4.0 Å scans (original Wilson-Popelier range). Only 25 molecules are listed because malonaldehyde is absent due to methylene hydrogens being too close together, leading to an error in GAUSSIAN09[3].

Molecule	Def A / kJ mol^{-1}	Def B / Å^{-1}	Def RMSE / kJ mol^{-1}	Str A / kJ mol^{-1}	Str B / Å^{-1}	Str RMSE / kJ mol^{-1}
Carbon Dioxide	50226.8	2.41	0.9	44343.2	2.31	1.4
Formaldehyde	74280.4	2.64	2.8	29607.3	2.14	2.8
Ethanal	85386.6	2.70	3.6	31820.3	2.16	2.5
Formamide	109177.6	2.83	5.3	36759.4	2.23	2.2
Formic Acid	63170.8	2.55	1.5	35924.5	2.21	2.1
Propanone	96476.2	2.78	4.2	32735.4	2.18	2.3
Acetamide	118249.5	2.97	6.1	31046.9	2.23	1.5
Acetic Acid	119297.1	2.89	5.7	37427.1	2.25	2.1
Urea	90915.8	2.67	5.7	42310.5	2.25	0.8
Carbamic Acid	126188.6	2.91	6.6	42809.0	2.30	1.5
Carbonic Acid	48437.4	2.42	0.4	38530.7	2.24	1.4
Carbonyl Fluoride	57501.8	2.52	0.7	39988.2	2.27	1.9
Cyclopropenone	138081.9	2.95	6.5	39004.6	2.24	2.8
Difluoroacetic Acid	34761.2	2.31	0.7	23786.8	2.06	0.9
Ethenone	54959.0	2.46	0.4	57633.5	2.43	1.0
Fluoroacetic Acid	111847.2	2.86	5.2	32701.0	2.18	2.8
Fluoroacetone	81906.3	2.75	3.8	24976.3	2.09	2.8
Fluorocarboxylic Acid	52660.9	2.47	0.6	39265.7	2.25	1.6
Propanal	119293.3	2.83	3.4	27596.1	2.10	1.5
Propiolaldehyde	72743.6	2.63	2.4	32892.4	2.18	2.6
TFA	93635.1	2.68	7.9	26523.5	2.10	1.6
Methyl Methanoate	65095.3	2.57	1.8	36347.7	2.22	2.0
Formyl Fluoride	64968.2	2.57	1.4	36404.3	2.23	2.3
Propenal	29005.0	2.27	13.0	33011.9	2.18	1.8
DMF	124350.1	2.91	6.2	36318.7	2.22	2.2

Table S7. Values for the A and B parameters along with the RMSEs of their fits to the deformation and steric energy data for the carbon atom in the 2.7-4.5 Å scans. Blanks due to errors or non-exponential behaviour.

Molecule	Def A / kJ mol^{-1}	Def B / Å^{-1}	Def RMSE / kJ mol^{-1}	Str A / kJ mol^{-1}	Str B / Å^{-1}	Str RMSE / kJ mol^{-1}
Carbon Dioxide				2603.5	1.47	0.7
Formaldehyde	-77.1	0.67	1.0	1647.9	1.46	0.6
Ethanal	-451.1	1.35	0.5	39,335.5	2.63	0.7
Formamide	-106,4391.9	4.00	2.9	18,901.0	2.72	0.1
Formic Acid	-1919.9	1.39	0.4	4227.9	2.39	0.1
Propanone	-3725.5	1.95	0.6	217,376.2	3.21	1.7
Acetamide				4.3e+08	6.49	2.3
Acetic Acid	-14,537.1	2.11	0.6	1.5e+13	1.06	3.5
Urea				101.2	0.93	0.1
Carbamic Acid	-665,101.5	3.42	4.5	413,263.2	3.39	2.7
Carbonic Acid	-16,330.3	1.93	0.8	9583.0	2.02	0.2
Methyl Methanoate	-1862.4	1.44	0.3	13192.0	2.82	0.0
Formyl Fluoride	-1862.9	1.31	0.2	3077.9	2.31	0.1
Propanal	-2095.0	1.69	0.6	39,804.4	2.54	0.7
Propenal	-1017.2	1.63	1.1	29,718.5	2.46	1.1
DMF				1,795,789.8	4.45	0.7
Malonaldehyde	-756.6	1.28	0.6	11753.9	2.11	0.1
Ethenone	67,399.0	3.24	0.8	155,873.5	3.14	0.8
Cyclopropenone				9.9e+26	2.23	11.6
Carbonyl Fluoride	-5375.6	1.36	2.5	2067.6	1.42	0.9
Fluoroacetic Acid	-5781.7	1.69	0.5	4.1e+09	7.44	2.4
Fluoroacetone	-1073.8	1.34	0.2	23,501.2	2.34	0.1
Fluorocarboxylic Acid	-13,119.9	1.80	0.9	5236.0	1.80	0.4
TFA	-6527.6	1.45	0.9	8498.3	2.22	0.1
Difluoroacetic Acid	-4758.5	1.45	0.9	5631.2	2.14	0.2
Propiolaldehyde				10,820.9	2.06	0.3

Table S8. Values for the A and B parameters along with the RMSEs of their fits to the deformation and steric energy data for the carbon atom in the 2.3-4.5 Å scans (original Wilson-Popelier range). Blanks due to errors or non-exponential behaviour.

**Molecule**	**Def A / kJ mol^{-1}**	**Def B / Å^{-1}**	**Def RMSE / kJ mol^{-1}**	**Str A / kJ mol^{-1}**	**Str B / Å^{-1}**	**Str RMSE / kJ mol^{-1}**
Carbon Dioxide				4990.2	1.68	1.7
Formaldehyde	-18.3	0.28	2.5	4129.9	1.76	1.5
Ethanal	-81.4	0.83	1.7	20,421.4	2.41	0.9
Formamide	-32,171.8	2.77	3.1	26,334.9	2.84	0.1
Formic Acid	-2632.6	1.49	0.9	6931.5	1.78	1.9
Propanone	-575.8	1.35	2.0	39,165.4	2.61	2.1
Acetamide				240,436.1	3.78	2.4
Acetic Acid	-7779.1	1.90	1.3	911,547.4	4.49	3.4
Urea				470.2	1.43	1.1
Carbamic Acid	-74,994.8	2.65	5.2	68,740.3	2.76	3.0
Carbonic Acid	-20,968.6	2.01	1.1	13,332.2	2.13	0.6
Methyl Methanoate	-2659.3	1.55	0.8	20,153.9	2.97	0.1
Formyl Fluoride	-2015.7	1.34	0.4	7004.9	2.60	0.2
Propanal	-281.1	1.06	2.9	17,409.1	2.25	1.2
Propenal	-60.4	0.80	2.8	16,640.1	2.26	1.2
DMF				100,528.1	3.41	0.8
Malonaldehyde	-142.4	0.78	3.5	9173.2	2.03	0.4
Ethenone	53,443.4	3.14	0.8	58,413.5	2.79	1.0
Cyclopropenone						
Carbonyl Fluoride	-9282.4	1.53	4.7	3641.5	1.60	1.6
Fluoroacetic Acid	-5550.8	1.67	0.5	322,076.0	4.02	2.3
Fluoroacetone	-656.7	1.18	1.2	19,146.5	2.27	0.3
Fluorocarboxylic Acid	-17,053.6	1.89	1.4	7455.5	1.92	0.8
TFA	-46,640.9	2.13	25.8	10,478.7	1.56	1.5
Difluoroacetic Acid	-245,410.1	2.88	20.9	7701.1	2.25	0.3
Propiolaldehyde				11,388.8	2.08	0.3

Occasionally, fits using our scan range resulted in extremely high A and B values due to the steric energy becoming negative at large interatomic separations, which would imply that the steric interaction becomes attractive at those distances. Two examples are shown in Table S9, where rounding the charge differently by using a ceiling function instead of a floor function, or using the next (meaning $\lfloor N \rfloor$ instead of $\lfloor N \rfloor + 1$) ionisation potential (actually energy) led to more reasonable parameter values and lower RMSE.

Table S9. Effect of using different values of charge N when calculating charge transfer for carbonyl carbon of fluoroacetic acid and cyclopropenone.

Molecule	Atom/Ion	Steric A / kJ mol^{-1}	Steric B / Å^{-1}	RMSE / kJ mol^{-1}
Fluoroacetic acid	C^+ (original)	4.1x10^{9}	7.44	2.4
	C^{2+}	68,528.9	2.55	1.5
	C	-1138.8	1.35	0.4
Cyclopropenone	C^+ (original)	9.9x10^{26}	22.26	11.6
	C	353.7	1.28	0.7
	C^{2+}	-11.1	-0.31	9.1

While unphysical, the phenomenon of negative steric energies has been observed previously[2] with hydrogen atoms at distances smaller than 2.7 Å in relaxed ammonia dimer scans, where it was attributed to changing geometries during the scan. More similar to the current work, the data in that work[2] for a frozen methane dimer scan seems to show negative steric energies for the carbon atom at distances greater than around 3.5 Å although this is not mentioned in their text[2]. In the current work, where only frozen scans were performed, the negative steric energies occur for 12 of the 26 molecules studied, which are listed in Table S10. Currently, we can only offer a qualitative description of this phenomenon. In cases where the steric energy does not become significantly negative, such as ethanal and formamide, this could be due to numerical instability. More extreme cases such as cyclopropenone tend to occur when there is poorer contact between the carbonyl carbon atoms. However, poor contact does not necessarily lead to negative steric energies. Perhaps a way of quantifying the contact between atoms such as the interatomic surface area should be accounted for along with charge transfer in a further correction for steric energy.

Table S10. Distance at which the carbonyl carbon atoms exhibit negative steric energy. The most negative energy observed across the scan range is also given.

Molecule	Distance beyond which steric energy is negative / Å	Largest negative steric energy / kJ mol^{-1}
Ethanal	4.1	-0.9
Formamide	4.4	-0.1
Propanone	3.7	-2.2
Acetamide	3.2	-3.1
Acetic Acid	3.0	-4.5
Carbamic Acid	3.6	-3.6
Propanal	4.2	-0.6
Propenal	4.1	-1.4
DMF	3.5	-1.0
Ethenone	3.9	-1.1
Cyclopropenone	2.9	-14.2
Fluoroacetic Acid	3.1	-3.1

To allow for a fair comparison of transferability for carbon deformation and steric parameters, Figure 5b in the main text only contains molecules for which an exponential deformation energy curve could be fitted; the other molecules' deformation energy data displayed non-exponential behaviour. Figure S3b shows the transferability of the steric parameters for the carbon atom in all 26 molecules.

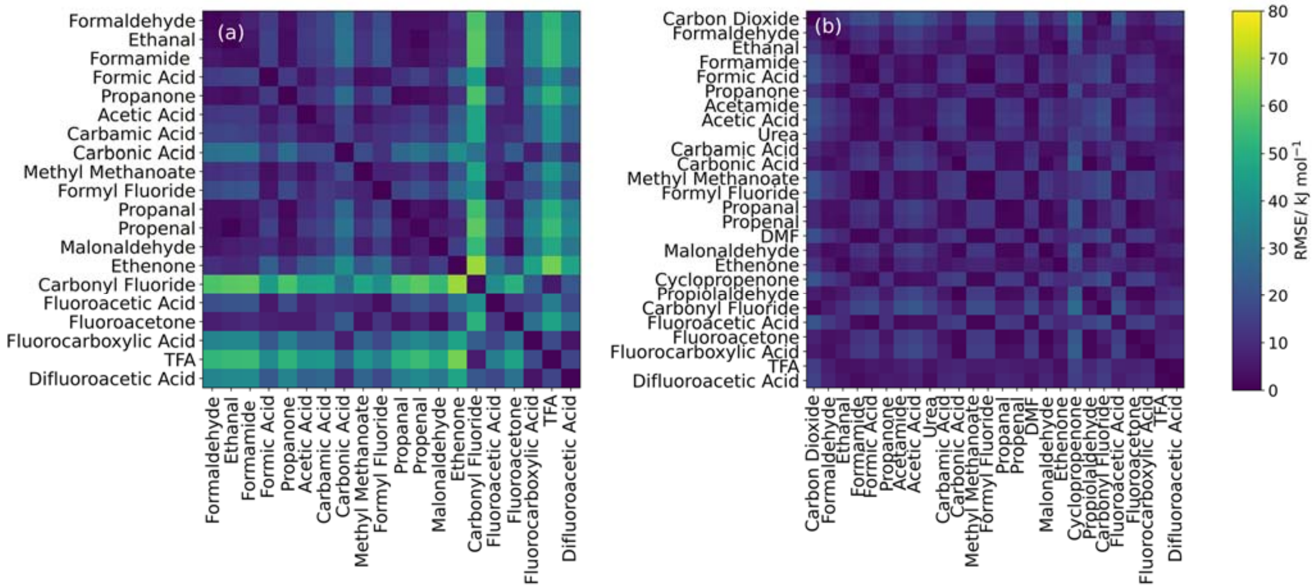

Figure S3. Heatmaps showing the transferability of A and B parameters of the carbonyl carbon atom for (a) deformation energy in 20 molecules and (b) steric energy in all 26 molecules. Diagonal cells represent the RMSE of the fitted Buckingham potential and the data it was fitted to, while off-diagonal cells represent transferability RMSEs of the Buckingham curve plotted using the A and B values of the molecule in the row compared to the data of the molecule in the column.

4. Properties

Wilson and Popelier performed dimeric scans of noble gases and small molecules, and fitted deformation energy to Buckingham potentials[1]. Figure S4 shows a plot of A against B from these fits. The lower R^2 value here compared to those of >0.9 in Figures 6 and 7 in the main text is due to the larger variety of systems studied in the work of Wilson and Popelier.

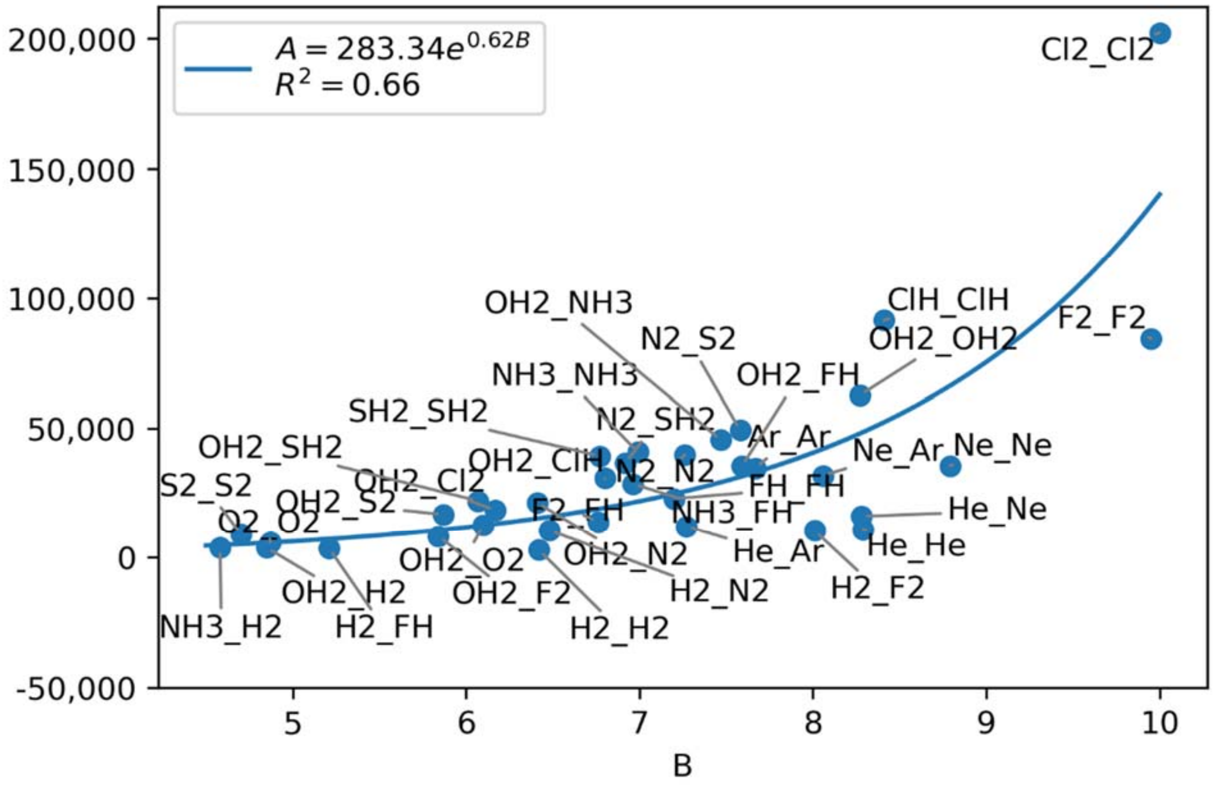

Figure S4. Relationship of A and B parameters from the work of Wilson and Popelier[1].

Plots of A and B against the molecular (monomeric) dipole moment and volume of a topological atom were created as a first attempt to link the parameters to a physical property. These are shown in Figure S5 (oxygen) and Figure S6 (carbon) for the dipole moment, and in Figure S7 for the volume. Monomeric molecular dipole moments were calculated using GAUSSIAN09[3] at the B3LYP/aug-cc-pVTZ level of theory. Topological atom volumes were calculated using the gs30 quadrature and Proaim method of the program[4] AIMAll19.

The figures in this section demonstrate the possible links between A and B that were examined in our current study. Figure S4 demonstrates that the exponential relationship between A and B is not limited to our study, the specific systems we studied, or indeed to homo-atomic interactions because it includes some hetero-atomic interactions. Figures S5, S6, and S7 show that there is no straightforward relationship between A and B, and the dipole moment or the volume of topological atoms. Nevertheless, there are weak correlations between oxygen deformation A and B and molecular dipole moment in Figures S5a and S5c due to the connection between molecular dipole moment and charge transfer.

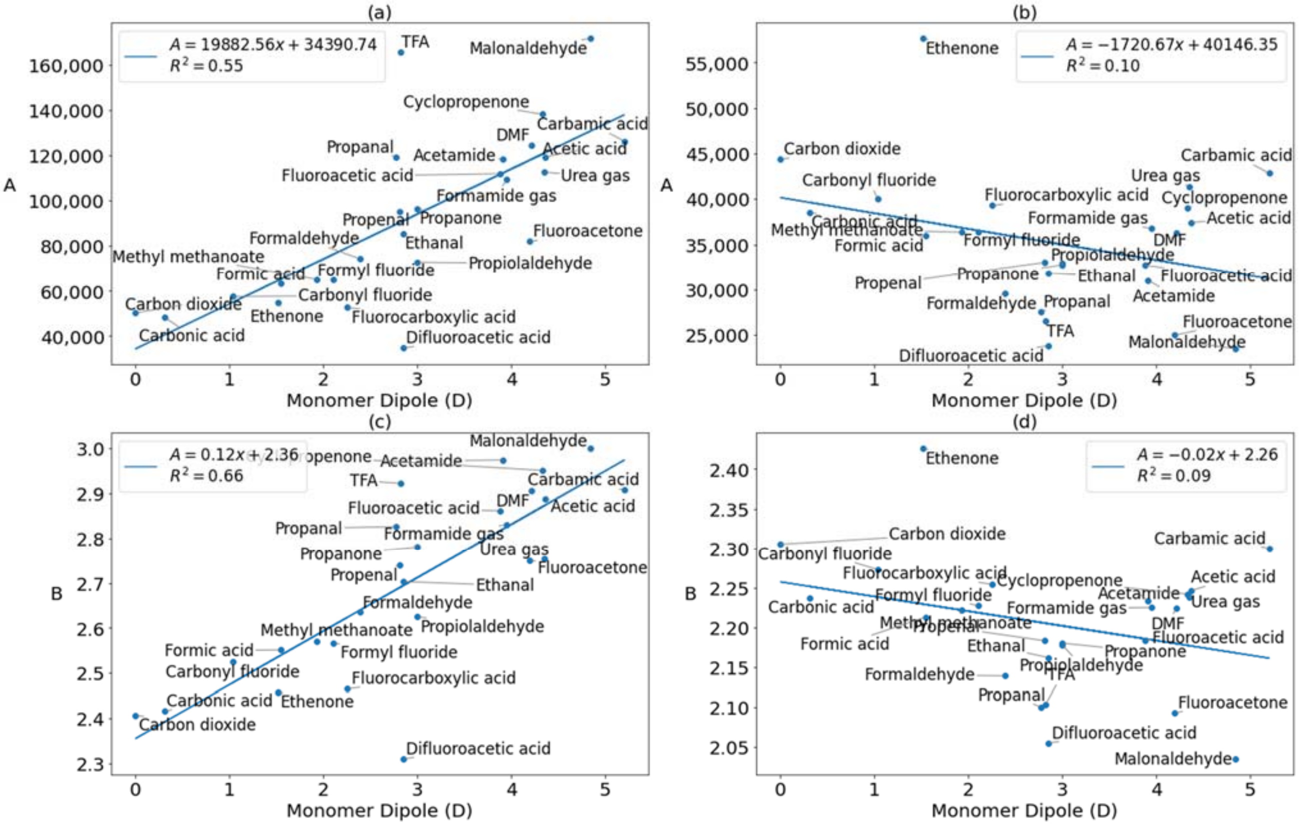

Figure S5. Plots of oxygen A and B parameters against molecular dipole moment for (a) deformation A, (b) steric A, (c) deformation B, and (d) steric B.

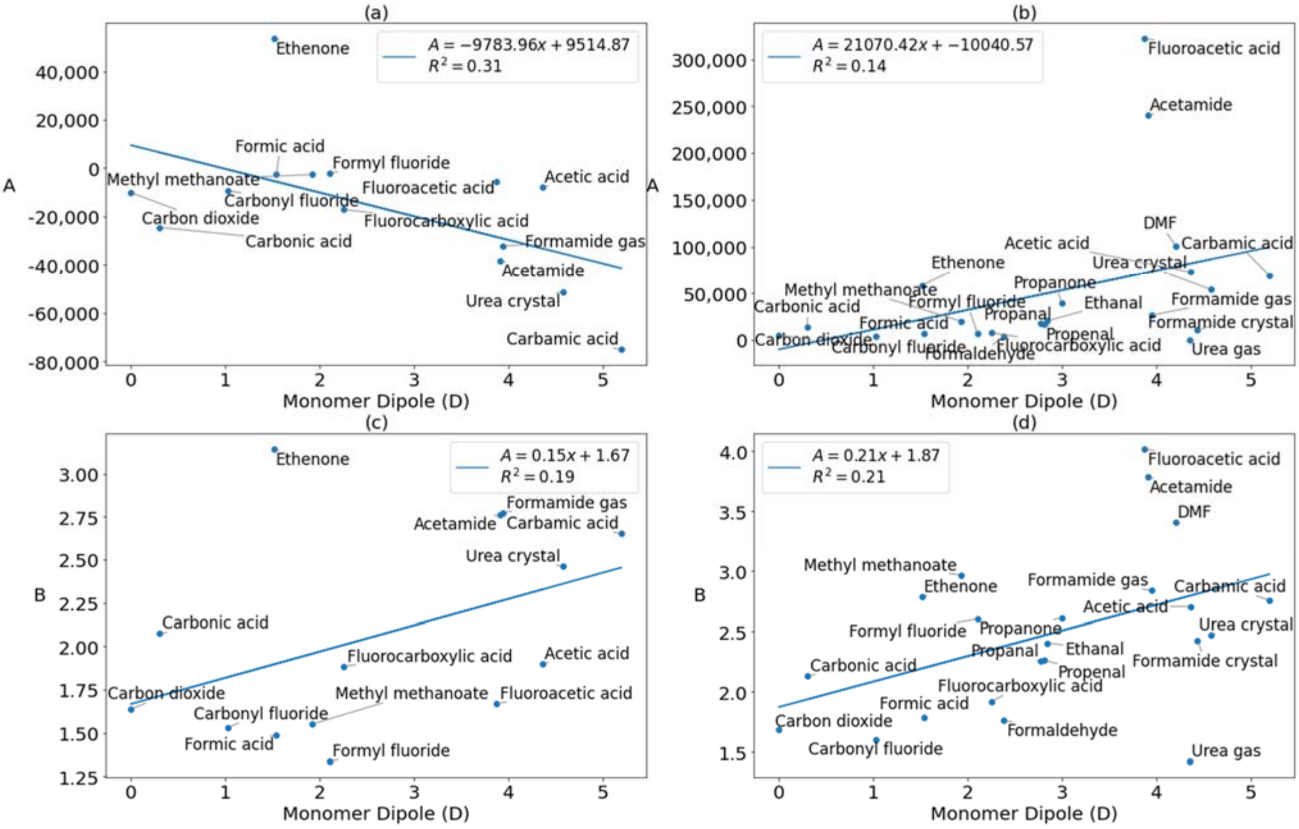

Figure S6. Plots of carbon A and B parameters against molecular dipole moment for (a) deformation A, (b) steric A, (c) deformation B, and (d) steric B.

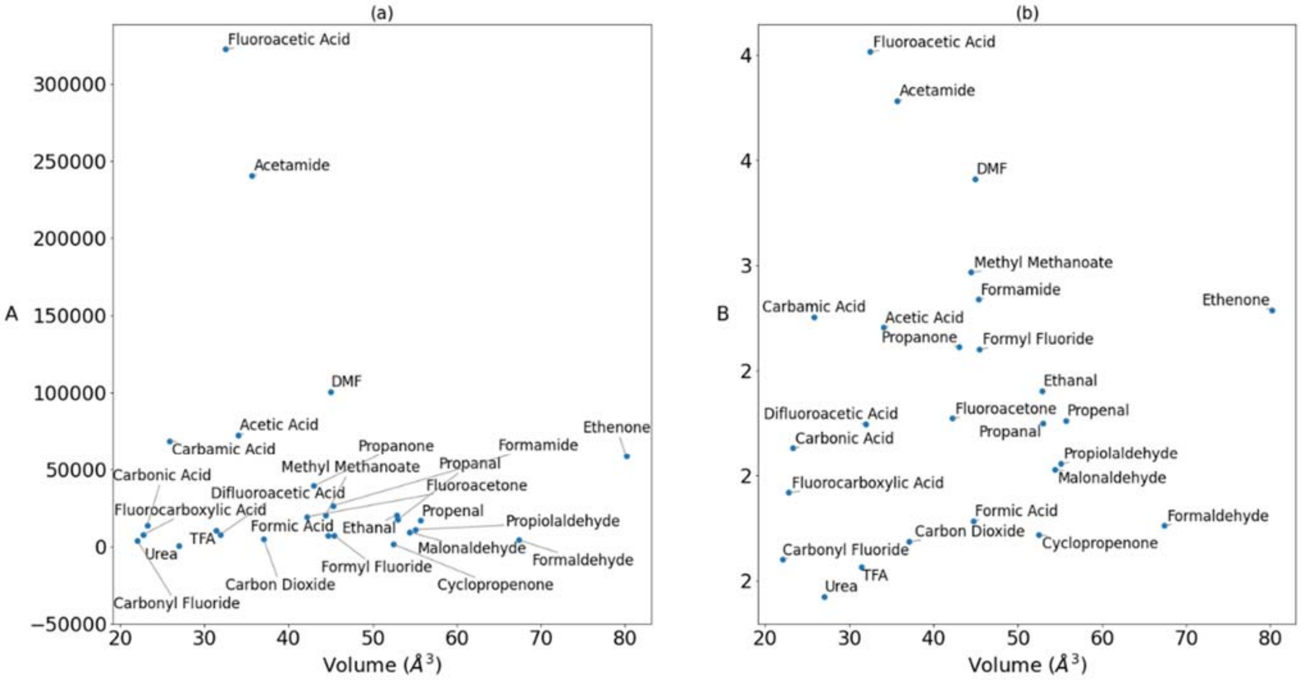

Figure S7. Example plots of (a) parameter A, and (b) parameter B against atomic volume at 2.6 Å intermolecular separation for carbon steric energy.

References

1. Wilson, A. L.; Popelier, P. L. A., Exponential Relationships capturing Atomistic Short-range Repulsion from the Interacting Quantum Atoms (IQA) Method *J. Phys. Chem. A* **2016,** *120*, 9647-9659.

2. Gallegos, M.; Costales, A.; Martín Pendás, Á., Energetic Descriptors of Steric Hindrance in Real Space: An Improved IQA Picture. *ChemPhysChem* **2021,** *22* (8), 775-787.

3. GAUSSIAN09 Revision B.01, M. J. Frisch, G. W. Trucks, H. B. Schlegel, G. E. Scuseria, M. A. Robb, J. R. Cheeseman, G. Scalmani, V. Barone, B. Mennucci, G. A. Petersson, H. Nakatsuji, M. Caricato, X. Li, H. P. Hratchian, A. F. Izmaylov, J. Bloino, G. Zheng, J. L. Sonnenberg, M. Hada, M. Ehara, K. Toyota, R. Fukuda, J. Hasegawa, M. Ishida, T. Nakajima, Y. Honda, O. Kitao, H. Nakai, T. Vreven, J. A. Montgomery, Jr., J. E. Peralta, F. Ogliaro, M. Bearpark, J. J. Heyd, E. Brothers, K. N. Kudin, V. N. Staroverov, R. Kobayashi, J. Normand, K. Raghavachari, A. Rendell, J. C. Burant, S. S. Iyengar, J. Tomasi, M. Cossi, N. Rega, J. M. Millam, M. Klene, J. E. Knox, J. B. Cross, V. Bakken, C. Adamo, J. Jaramillo, R. Gomperts, R. E. Stratmann, O. Yazyev, A. J. Austin, R. Cammi, C. Pomelli, J. W. Ochterski, R. L. Martin, K. Morokuma, V. G. Zakrzewski, G. A. Voth, P. Salvador, J. J. Dannenberg, S. Dapprich, A. D. Daniels, Ö. Farkas, J. B. Foresman, J. V. Ortiz, J. Cioslowski, and D. J. Fox, GAUSSIAN09, revision D.01. *Gaussian, Inc., Wallingford CT, USA*, 2013.

4. Keith, T. A. AIMAll version 19, *TK Gristmill Software, Overland Park, Kansas, USA* **2019**.
